# Fecal metabolomics and gut microbiota profiling uncover the protective role of probiotic-rich traditional fermented sour soup (Guizhou Hongsuantang) against alcoholic liver damage

**DOI:** 10.3389/fnut.2026.1736948

**Published:** 2026-06-10

**Authors:** Xu Rui, Wu Ruijia, Bao Aiming, Qin Weijun, Chen Xingxing, Zhang Xiaoyong, Gao Xiangyang

**Affiliations:** 1 Guangdong Provincial Key Laboratory of Nutraceuticals and Functional Foods, College of Food Science, South China Agricultural University, Guangzhou, China; 2 Guangdong-Guizhou Science and Technology Collaboration Key Laboratory of Fermented Foods, Anshun, China; 3 Guizhou Nanshanpo Food Processing Co., Ltd., Anshun, China; 4 University Joint Laboratory of Guangdong Province, Hong Kong and Macao Region on Marine Bioresource Conservation and Exploitation, College of Marine Sciences, South China Agricultural University, Guangzhou, China

**Keywords:** bioactive compounds, Guizhou Hongsuantang, gut microbiota, hepatoprotection, HMDB, LIPID MAPS, lipid metabolism, metabolite

## Abstract

**Objective:**

This study investigates the metabolic mechanisms underlying the hepatoprotective effects and mitigation of alcohol-induced impacts of bacterial strains (*Lactobacillus plantarum* LP and *Lactobacillus paracasei* H2) isolated from “Guizhou Hongsuantang.”

**Methods:**

In order to identify changes in microbial composition, fecal metabolites and metabolic pathways linked to probiotic intervention, the study uses integrated gut microbiota analyses and metabolomics such as 16S rDNA sequencing, UHPLC-MS and functional predictions.

**Results:**

Pathway enrichment analysis revealed significant modulation of key metabolic pathways, particularly those associated with lipid metabolism such as steroid hormone biosynthesis and arachidonic acid metabolism as well as amino acid metabolism, membrane transport and bile secretion. These pathways are critical for regulating inflammation, oxidative stress and detoxification processes, which are commonly impaired during liver injury or alcohol-induced stress. Further metabolite classification identified a predominance of lipids, fatty acids, and organic acids with remarkable enrichment in subclasses such as fatty acyls, eicosanoids, isoprenoids and glycerophospholipids all of which are implicated in liver protection, energy metabolism and cellular repair. The intervention was associated with levels of microbial-derived metabolites and secondary bioactive compounds, including flavonoids and macrolides, suggesting an interaction between host metabolism and gut microbiota. Differential analysis across experimental groups revealed dose-dependent effects, with high-dose intervention (Group G) is correlated with the most substantial metabolic shifts. These findings clarify the gut-liver axis-related metabolic mechanisms of probiotic-rich “Guizhou Hongsuantang” in protecting against alcoholic liver damage.

**Conclusion:**

These findings provide a scientific basis for the development of probiotic-based functional fermented foods derived from traditional ethnic foods and offer a promising approach to reducing alcohol-induced hepatic injury and advancing the modernization of traditional ethnic fermented foods.

## 1 Introduction

Alcoholic beverages are widely consumed across various cultures and often integrated into social traditions. With the increasing pace of modern society and more frequent social activities, alcohol consumption has become a common part of daily life for many people. Alcohol drinking is a common practice worldwide, with around 2.4 billion people identified as drinkers, including 960 million considered heavy consumers (1). According to the 2018 Global Report on Alcohol and Health by the World Health Organization (WHO), high levels of alcohol consumption contribute to roughly 3 million deaths each year, representing 5.3% of all global fatalities and are implicated in 47.9% of deaths related to cirrhosis. Hangovers, which typically involve symptoms like headaches, fatigue, and nausea, can hinder mental performance and daily activities (2). Frequent alcohol drinking may contribute to progressive liver damage (3). Neuroscience studies have identified specific brain regions impacted by alcohol, offering insight into how it impairs reaction time (4). Moreover, researchers have explored the duration of these impairments following alcohol intake, with a focus on understanding their persistence over time (5).

Liver is uniquely equipped to convert cholesterol into bile acids, which facilitate nutrient, drug, and metabolite absorption and transport via enterohepatic circulation. These interconnected pathways of glucose, lipid, and energy metabolism are tightly regulated by hormones and nutrients to maintain metabolic homeostasis. Disruptions in these regulatory systems can contribute to metabolic disorders, including chronic liver diseases, alcoholic fatty liver disease, non-alcoholic fatty liver disease (NAFLD), non-alcoholic steatohepatitis (NASH), insulin resistance, diabetes, and obesity (6). Excessive drinking can lead to liver damage, as indicated by elevated blood levels of enzymes such as alanine transaminase (ALT), aspartate aminotransferase (AST) and lactate dehydrogenase (LDH), along with the buildup of lipids in the liver, resulting in alcoholic fatty liver disease (7–12). Liver function progressively declines as alcoholic liver disease (ALD) advances which is a major contributor to cirrhosis development (7, 10). Three primary mechanisms are thought to underlie alcoholic liver injury such as: toxicity from acetaldehyde; generation of reactive oxygen species (ROS) or oxidative stress through metabolism and an immune response that induces oxidative stress in liver cells (9, 13–16).

In recent years, probiotics and their fermentation products have attracted widespread attention due to their potential roles in regulating intestinal flora, enhancing immunity and anti-oxidation. Lactic acid bacteria, as one of the common types of probiotics, are not only widely used in the production of fermented foods, but have also been proven to have certain effects in improving alcoholic liver damage (17–20). In particular, during the fermentation process, lactic acid bacteria can synergize with a variety of plant raw materials to produce a variety of biologically active metabolites, such as polyphenols, short-chain fatty acids and antioxidant enzymes, which provide a theoretical and practical basis for the development of functional fermented foods. Ethanol also affects gut microbial composition by altering the physical and chemical environment of the intestine. In ethanol-fed mice, bacterial diversity was markedly reduced (21). Clinical studies on patients with alcoholic cirrhosis revealed a microbial imbalance marked by a deficiency in *Lactobacillus* species and an overgrowth of *Candida* fungi (22). Research has shown that LAB can be effective in treating alcohol-related liver damage, offering multi-faceted benefits without notable side effects (23, 24).

“Hongsuantang” is a traditional fermented food of ethnic minorities in Guizhou (25). It is rich in a variety of beneficial bacteria and plant ingredients and has the effect of improving liver lipid metabolism. This study aims to evaluate the protective effects of lactic acid bacteria isolated from traditional “Guizhou Hongsuantang” on alcoholic liver injury in mice by investigating their roles in ethanol metabolism, liver function improvement and antioxidant activity. To uncover the underlying mechanisms, the study employs integrated gut microbiota analyses and metabolomics including 16S rDNA sequencing, UHPLC-MS and functional predictions to identify changes in fecal microbial composition, metabolites and metabolic pathways associated with probiotic intervention.

## 2 Materials and methods

### 2.1 Experimental animals

Male Kunming mice (4 weeks old, 18–22 g) were obtained from Zhuhai Baishitong Biotechnology Co., Ltd. (Zhuhai, China; production license No. SCXK 2020-0051). Animals were housed in a barrier system at the experimental animal center of South China Agricultural University under controlled environmental conditions (temperature: 25 ± 1°C; relative humidity: 60 ± 5%) with a 12 h light/12 h dark cycle. Mice were provided *ad libitum* access to standard chow and water throughout the experimental period. All animal procedures were conducted in accordance with institutional guidelines and were approved by the Experimental Animal Ethics Committee of South China Agricultural University (Approval No. 2024B217).

About 50 mice were acclimatized for 7 days and randomly divided into 5 groups of 10 mice each. Mouse samples were divided based on intervention status: group A (blank group), group B (model group), group C (positive control group), group F (low dose intervention group) and group G (high dose intervention group). Mice in the blank control group and model group were administered 5 mL/kg of physiological saline (calculated by body weight) by gavage daily. Mice in the positive control group were administered 5 mL/kg of silymarin solution (concentration of 10 mg/mL) by gavage. Mice in the low-dose group and high-dose group were administered 5 mL/kg and 15 mL/kg of mixed-culture fermented red sour soup sample solution by gavage, respectively. Within 30 min after the last administration, all mice in the treatment groups, except for the blank control group, were administered 10 mL/kg of Hongxing Erguotou liquor (53% vol) by gavage. The blank control group was administered an equal volume of physiological saline by gavage. The gavage was performed for 14 days, from 8:00 to 9:00 a.m. daily. During the experiment, the body weight of the mice was measured daily, and the daily dosage was adjusted according to the changes in body weight.

Mice were deeply anesthetized with 1.25% tribromoethanol until loss of reflexes was confirmed, followed by cervical dislocation as a secondary method to ensure death, in accordance with AVMA guidelines and institutional IACUC approval.

### 2.2 Reagents, culture media, and instruments

The experimental procedures utilized several reagents, including Red Star Erguotou (53%) (Beijing Red Star Co., Ltd., China), Ligalon Silymarin Capsules (Dr. Ma Pharmaceutical Factory, Germany), Sodium chloride (Guangzhou Licheng Industrial Co., Ltd., China) and analytical-grade Anhydrous Ethanol (Tianjin Damao Chemical Reagent Factory, China). Samples of traditional fermented red sour soup (Hongsuantang) were generously provided by local farmers from the Qiannan Buyi and Miao Autonomous Prefecture, Guizhou Province, China.

MRS agar and MRS broth media were purchased from Guangdong Huankai Biotechnology Co., Ltd. The composition of the MRS agar medium was as follows: 10 g tryptone, 5 g yeast extract, 20 g glucose, 2 g potassium dihydrogen phosphate, 5 g sodium chloride, 2 g dipotassium hydrogen phosphate, 1 g citric acid, 0.1 g magnesium sulfate heptahydrate (MgSO_4_⋅7H_2_O), and 15 g agar per 1,000 mL of distilled water. The medium was sterilized by autoclaving at 121°C for 20 min. The MRS broth medium had the same composition as the agar medium, excluding agar, and was similarly sterilized at 121°C for 20 min. Lactic acid bacteria (LAB) were isolated from “Guizhou Hongsuantang” using the serial tenfold dilutions (10^−1^–10^−10^) with 0.85% sterile physiological saline. Aliquots (0.1 mL) from each dilution were spread onto MRS plates and incubated under anaerobic conditions at 37°C for 24–48 h. After incubation, colonies displaying distinct morphological features, including differences in size, shape and pigmentation, were selected for further characterization. Purification of LAB isolates was achieved by repeated streaking of selected colonies onto fresh MRS agar plates to obtain single, well-isolated colonies. This sub culturing procedure was performed multiple times until consistent and uniform colony morphology indicated purity. Cultures were centrifuged at 10,000 rpm for 10 min at 4°C. The bacterial pellets were washed three times with sterile saline (0.85%), and then resuspended to a final concentration of 1 × 10*8* CFU/mL. The bacterial suspension was inoculated into prepared red sour soup at a 3% (v/v) inoculation rate. A 1:1 mixture of *L. plantarum* LP and *L. paracasei* H2 was fermented at 28 ± 1°C for 15 days. For oral administration, the fermented red sour soup was pasteurized at 67°C for 20 min, cooled to room temperature and centrifuged at 7,000 rpm for 15 min at 4°C. The precipitate was discarded and the supernatant was collected and used for gavage.

Sample processing was performed using instruments including culture shaker (Model: HYL-X; Zhejiang Taicang Qiangwen Experimental Co., Ltd., China), double-person single-sided clean bench (Model: SW-CJ-2F; Lichen Technology Co., Ltd., China), vertical high-pressure steam sterilizer (Model: LS-50GH; Zhejiang Jiangyin Binjiang Medical Equipment Co., Ltd., China), water bath (model: HH-4; Changzhou Aohua Instrument Co., Ltd., China) and PCR thermal cycler (Model: A100; Hangzhou Langji Scientific Instrument Co., Ltd., China). Metabolite analysis was conducted using the ultra-high-performance liquid chromatography (UHPLC) system (Model: Acquity I-Class PLUS; Waters, United States) equipped with a C18 reversed-phase column (150 mm length × 2.0 mm inner diameter, 2.0 μm particle size) and high-resolution mass spectrometer (Model: Xevo G2-XS QTOF; Waters, United States).

### 2.3 Sequencing analysis

#### 2.3.1 Sanger sequencing of isolates for strain identification

Genomic DNA was extracted from purified bacterial cultures using TIANamp Genomic DNA Kit (TIANGEN, China), following the manufacturer’s instructions. The extracted DNA served as the template for amplification of the 16S rDNA gene using polymerase chain reaction (PCR). Universal primers 27F (5′-AGAGTTTGATCCTGGCTCAG-3′) and 1492R (5′-TACGGTTACCTTGTTACGACTT-3′) were used for amplification. The PCR reaction mixture (total volume: 50 μL) consisted of 25 μL of 2 × Taq Master Mix (Dye Plus), 1.5 μL each of the forward and reverse primers, 2.0 μL of DNA template (20 ng/μL), and nuclease-free water to a final volume of 50 μL. The PCR cycling conditions were as follows: initial denaturation at 94°C for 5 min; 34 cycles of denaturation at 94°C for 30 s, annealing at 58°C for 30 s, and extension at 72°C for 90 s; followed by a final extension at 72°C for 10 min. PCR products were verified by electrophoresis on a 1% agarose gel and subsequently sent to Guangzhou Tianyi Biotechnology Co., Ltd. for sequencing. The resulting sequences were submitted to the GenBank database of the National Center for Biotechnology Information (NCBI), and homology analysis was performed using the Basic Local Alignment Search Tool (BLAST).

#### 2.3.2 Illumina-based 16S rRNA gene sequencing for community profiling

Fecal samples for the study were collected from control, model, and intervention group mice. Approximately 1–3 grams of each sample was placed into a test tube frozen to a -80°C freezer until further analysis. Total genomic DNA was extracted from mouse fecal samples using TIANamp Stool DNA Kit (TIANGEN, China) according to the manufacturer’s instructions. Following the extraction, primers targeting conserved regions were designed to amplify the desired genetic fragments. Sequencing adapters were ligated to the 5′ and 3′ ends of these primers to facilitate downstream sequencing. PCR was used to amplify the target regions, after which the resulting amplicons underwent purification to remove contaminants. The purified PCR products were then quantified and normalized to ensure uniform representation in the sequencing pool, forming the final sequencing library. Library quality was assessed through standard quality control procedures to confirm suitability for sequencing. Libraries that met quality criteria were subjected to high-throughput sequencing using the NovaSeq 6000 platform (Illumina, United States). These reads were exported in FASTQ format, which stores both the nucleotide sequence and the corresponding base quality scores for each read.

#### 2.3.3 NGS data preprocessing

Raw sequencing reads were initially quality-filtered based on nucleotide accuracy using Trimmomatic (v0.33) (26). Primer sequences were subsequently detected and removed with Cutadapt (v1.9.1) (27).

#### 2.3.4 ASV analysis

For ASV Analysis, DADA2 (28) method in QIIME2 (version 2020.06) was applied to de-noise sequences, generating ASVs. Conservative threshold for ASV filtration was 0.005%.

#### 2.3.5 Taxonomic annotation and community composition analysis

Feature sequences were taxonomically classified using a Bayesian classifier and BLAST algorithm, referencing the SILVA database (release 111; July 2012) (29). Taxonomic composition was assessed across multiple hierarchical levels, including phylum, class, order, family, genus, and species. QIIME was utilized to quantify the relative abundance of taxa within each sample. Visual representations, such as distribution histograms for each taxonomic level, were generated using appropriate R packages (30).

#### 2.3.6 Multivariate statistical analysis

To explore patterns of variation among individuals or groups, Principal Component Analysis (PCA) (31) was conducted, focusing on the major axes of variation derived from the distance matrix. A heat map was generated using R to illustrate their distribution across samples. Hierarchical clustering was employed to assess the similarity among samples and to evaluate community composition patterns across different taxonomic levels.

#### 2.3.7 Correlation network analysis

A correlation network was constructed by including only those associations with a correlation coefficient > 0.1 and a *p*-value < 0.05, ensuring both strength and statistical significance of the relationships.

#### 2.3.8 16 S Functional genes prediction

The functional composition of environmental microorganisms was predicted through PICRUSt2 (32). The significance of difference in function abundance between samples was evaluated by G-TEST (Number of annotated functional genes > 20) and Fisher (Number of annotated functional genes < 20) in STAMP. Threshold for significant difference was set as *P*-value smaller than 0.05. FAPROTAX was used to predict the ecological function by identifying the name of the genus and species of bacteria (33).

#### 2.3.9 Phenotypic prediction

The biolevel coverage of functional pathways was predicted using BugBase (34). First, for each sample in the biological dataset, the relative abundance of traits was estimated across the entire range of the threshold (0–1, increments of 0.01). After setting the threshold, BugBase generated the organism level traits prediction table containing the relative abundance of predicted traits.

### 2.4 Metabolomics analysis

#### 2.4.1 Sample collection and preparation

Mice Fecal samples (50 mg) were first diluted with 450 μL of phosphate-buffered saline (PBS) to prepare for extraction. The mixture was then subjected to intense mixing using a vortex mixer for 1 min to ensure thorough homogenization. Afterward, the sample was placed on ice for 5 min to allow the components to stabilize. The mixing process was repeated for another minute using the vortex mixer, followed by a 2-min incubation on ice. After this, the sample was centrifuged at 10,000 g for 10 min at 4°C to separate the solid matter from the liquid phase. The supernatant was carefully collected and subjected to further filtration using a centrifugal filter to remove any remaining particulate matter.

#### 2.4.2 Ultra-high-performance liquid chromatography (UHPLC) and mass spectrometry

The mobile phase consisted of two components: phase A consisted of 15 mM acetate and 10 mM tributylamine in water, while phase B was methanol. The flow rate was set at 0.3 mL per minute and the injection volume was 3 μL. The column temperature was maintained at 40°C during the analysis. The mass spectrometry detection was performed using a high-resolution mass spectrometer (Xevo G2-XS QTOF) system, operating in negative ionization mode and employing multiple reaction monitoring (MRM). The drying gas temperature was set to 250°C, and the nebulizing gas was at 2.0 L/min, with a drying gas flow rate of 10 L/min.

#### 2.4.3 Metabolite qualitative and quantitative analysis

Raw data obtained from the mass spectrometry analysis were processed using MassLynx V4.2 software for peak detection, alignment, and data cleaning. Identification of metabolites was performed using Progenesis QI software, which was cross-referenced with the online METLIN database, public repositories, and an in-house database (35). Additionally, theoretical fragmentation patterns were utilized for further confirmation of metabolite identities.

#### 2.4.4 Differential metabolites analysis

Metabolites were initially evaluated based on fold change (FC), variable importance in projection (VIP), and statistical significance (*p*-value). Metabolites with an FC ≥ 1 were retained for further consideration. For datasets with biological replicates, metabolites with variable importance in projection (VIP) ≥ 1 were considered to have a strong contribution to group discrimination and were therefore retained as candidate differential metabolites. In addition, univariate statistical analysis was performed using Student’s *t*-test to evaluate the significance of differences between groups. Metabolites with a *p*-value < 0.05 were considered statistically significant. Only metabolites meeting all applicable criteria (VIP ≥ 1 and *p*-value < 0.05, in the presence of biological replicates) were ultimately defined as differential metabolites and used for downstream analyses.

#### 2.4.5 KEGG (kyoto encyclopedia of genes and genomes), human metabolome database (HMDB) and IPID MAPS (lipid metabolites and pathways strategy) analysis

The metabolic pathways were analyzed by KEGG (Kyoto Encyclopedia of Genes and Genomes) database (36) and pathway enrichment analysis was performed using MetaboAnalyst. Statistical significance was determined using *p*-values with false discovery rate (FDR) correction. To interpret the biological relevance of the altered fecal metabolites associated with fermented sour soup intervention, only significantly enriched pathways (FDR < 0.05) were selected for further analysis and visualization. Human Metabolome Database (HMDB) (37) and IPID MAPS (Lipid Metabolites and Pathways Strategy) (38) were applied to analyze human related small molecular metabolites and lipidomics, respectively.

## 3 Results

### 3.1 Fermentation preparation and activity evaluation

A *Lactobacillus plantarum* strain (Accession No. PV931985) was isolated from naturally fermented persimmon vinegar after 6 months of incubation at room temperature. In addition, commercial strains of *Lactobacillus rhamnosus* and *Lactobacillus acidophilus* were obtained from Angel Yeast Co., Ltd. for comparative analysis. Red sour soup was prepared by screening *Lactobacillus plantarum* LP and *Lactobacillus paracasei* H2 for mixed fermentation. Previous experimental results from the same lab showed that fermented sour soup not only accelerated alcohol metabolism and shortened the duration of alcohol action, but also alleviated alcohol-induced liver damage, improved lipid metabolism, and enhanced liver antioxidant capacity.

### 3.2 Sequencing analysis results

#### 3.2.1 Raw data quality control

A total of 1,578,949 paired-end (PE) reads were generated from 20 individual samples. Following the quality control and assembly of the PE reads, 1,458,698 high-quality clean reads were retained. The number of clean reads per sample ranged from a minimum of 54,081 to an average of 72,935. The initial number of raw reads per sample ranged from 58,488 (G3) to 80,271 (F1), with most samples clustering around 80,000 reads, indicating consistent sequencing depth across the dataset with the exception of G3. After quality filtering, clean reads ranged from 54,081 (G3) to 75,134 (G1), showing high retention rates. The denoising step resulted in a marginal reduction in read counts, with denoised reads varying from 53,966 (G3) to 74,932 (G1), indicating minimal sequence loss. Merged reads showed a slightly larger drop, ranging from 50,011 (G3) to 70,556 (G1), as expected due to sequence overlap constraints. The final non-chimeric read counts, representing high-confidence, usable sequences, ranged from 44,752 (G3) to 64,870 (G1). On average, approximately 81% of raw reads were retained as non-chimeric reads.

#### 3.2.2 Read count at each taxonomic level

The read counts at various taxonomic levels for each sample, including Kingdom, Phylum, Class, Order, Family, Genus and Species were analyzed. For each sample, the number of reads consistently decreases from the Kingdom level to the Species level, with the highest counts observed at the Kingdom level and the lowest at the Species level. The read counts across different taxonomic levels exhibit a similar pattern for each sample, with only minor variations observed. In sample A1, 54,420 reads were assigned at the Kingdom level, with a slight reduction to 33,665 reads at the Species level. Similar trends were observed in all other samples, with sample B3 having the highest read counts at all taxonomic levels, ranging from 63,558 at the Kingdom level to 37,203 at the Species level.

#### 3.2.3 Taxonomic annotation summary

**FIGURE 1:**
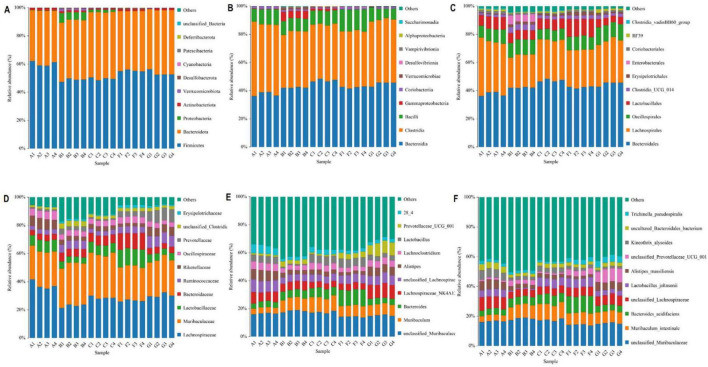
Histogram (pie chart) of taxonomic distribution. **(A)** Phylum; **(B)** Class; **(C)** Order; **(D)** Family; **(E)** Genus; **(F)** Species (*n* = 4).

The number of annotated species varied between samples, ranging from 189 in sample C1 to 229 in sample B2. Similarly, the annotation of higher taxonomic levels such as Family and Order exhibited greater variability, with the highest counts observed in sample B2, which had 65 annotations at the Family level and 44 at the Order level. This pattern reflects a broader taxonomic diversity and the varying complexity of the microbial communities across different samples (Figures 1A–F). The taxonomic composition of microbial communities across samples (A1 through G4) was analyzed at multiple taxonomic levels, revealing key patterns of abundance. At the phylum level, Bacteroidota and Firmicutes were the dominant taxa, with smaller proportions of Proteobacteria, Actinobacteriota, and other phyla, alongside a consistent presence of unclassified bacteria. At the class level, Bacteroidia and Clostridia dominated, followed by less abundant classes such as Saccharimonadia and Alphaproteobacteria. Order-level analysis showed Clostridiales and Bacteroidales as the most abundant, while other orders like Lactobacillales and Erysipelotrichales were also present. Family-level distribution highlighted variability, with Prevotellaceae, Lactobacillaceae, and Lachnospiraceae being more prominent in specific samples, indicating diverse microbial profiles. At the genus and species levels, variability increased, with *Lactobacillus*, *Bacteroides*, and *Muribaculum* being significant genera, and species such as *Lactobacillus johnsonii* and *Muribaculum intestinale* contributing notably. Unclassified genera and species were also present, underscoring the complexity and unidentified diversity within the microbiomes.

#### 3.2.4 Overview of microbial community structure across intervention groups

**FIGURE 2:**
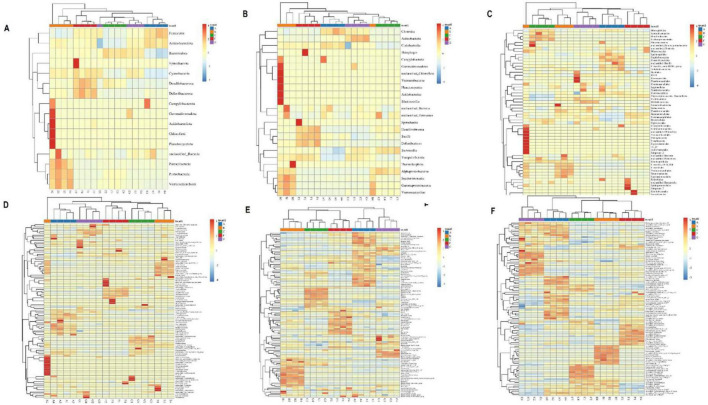
Taxonomic abundance cluster heat maps. **(A)** Phylum; **(B)** Class; **(C)** Order; **(D)** Family; **(E)** Genus; **(F)** Species (*n* = 4).

The relative abundance of dominant bacterial phyla across all samples, clustered by both taxa and intervention groups (A-G) are presented by heat maps (Figures 2A–F). Hierarchical clustering reveals distinct microbial community structures that group samples primarily by intervention. Samples from intervention group B formed a tightly clustered group, characterized by a pronounced enrichment of Actinobacteriota, Spirochaetota, and Campylobacterota, as indicated by strong red coloration in the corresponding cells. Conversely, Firmicutes and Bacteroidota, the most dominant phyla across the dataset, displayed more uniform distributions, though Firmicutes appeared slightly more abundant in group A samples. Phyla such as Proteobacteria, Patescibacteria, and Verrucomicrobiota showed relatively consistent, low-level abundance across most samples, while Cyanobacteria, Deferribacterota, and Planctomycetota exhibited sporadic enrichment in a few samples (such as C3 and F2). The clustering of samples from groups F and G indicated moderately similar microbial compositions, with occasional elevations in Gemmatiomonadota and Chloroflexi. The presence of unclassified bacteria and the distribution of rare phyla such as Acidobacteriota and Chloroflexi contributed to the within-group diversity, particularly in interventions C and G. Samples from intervention group B were distinguished by strong enrichment in Clostridia and Actinobacteria, particularly in samples B1, B2, and B3, where Clostridia exhibited the highest z-scores (dark red). Similarly, sample B3 showed localized enrichment of Coriobacteriia, suggesting a distinct microbial profile within this group.

Group F samples (F1-F4) clustered separately and exhibited moderate enrichment in Gemmatimonadetes, Bacteroidia, and Deferribacteres, while class Alphaproteobacteria showed notable presence in sample F3. Samples from group A were generally characterized by low or intermediate abundance across most taxa, though Thermoleophilia and Gammaproteobacteria appeared moderately enriched in A2 and A4, respectively. Group G (G1-G4) and C (C1-C4) samples showed more dispersed and heterogeneous distributions. For instance, Planctomycetes and Verrucomicrobiae were enriched in some G and C samples, while classes like Blastocatellia and unclassified_Bacteria exhibited sample-specific variation. A few rare classes, such as Vampirivibrionia and Saccharimonadia, showed localized increases (C4 and F4), but remained low overall. The heatmap illustrates the relative abundance of bacterial taxa at the order level across multiple samples subjected to different interventions (A, B, C, F, G). Rows represent bacterial orders, while columns denote individual samples clustered according to intervention groups, as indicated by the color-coded annotation bar at the top.

Hierarchical clustering of both bacterial taxa (rows) and samples (columns) demonstrates distinct clustering patterns among interventions, suggesting that microbial community structures vary in response to different conditions. Certain taxa, such as Monoglobales, Burkholderiales, and Bacteroidales, display higher relative abundances in specific interventions, whereas others (such as Clostridiales, Spirochaetales, Lactobacillales) are more enriched in alternative intervention groups. The clustering also highlights co-occurrence relationships among bacterial taxa, with closely related groups exhibiting similar abundance patterns across samples. Hierarchical clustering of both samples and taxa highlights intervention-specific microbial signatures. Distinct family-level shifts are evident, with certain families such as Lachnospiraceae, Ruminococcaceae, and Prevotellaceae exhibiting higher relative abundance in specific interventions, while others (such as Clostridiaceae, Erysipelotrichaceae, Bacteroidaceae) dominate under different conditions. Clustering of samples further indicates that microbial communities are more similar within the same intervention groups than across different interventions, suggesting intervention-dependent restructuring of the microbiota. Hierarchical clustering of both genera and samples reveals distinct intervention-driven community structures. Samples within the same intervention group cluster more closely together, reflecting similar microbial profiles, whereas separation across groups indicates intervention-specific effects on microbial composition. Several genera, including Anaerococcus, Prevotella, Streptococcus, Lactobacillus, and Bacteroides, show higher relative abundance under specific interventions, suggesting differential microbial enrichment. Conversely, other taxa (such as Clostridium sensu stricto, Ruminococcus, and Eubacterium) exhibit decreased representation under certain conditions.

The clustering of microbial communities across different taxonomic levels was visualized through heatmaps, revealing intervention-specific patterns in microbial composition (Figures 2A–F). At the phylum level, Firmicutes and Bacteroidota dominated, with samples like B1 and B2 showing higher relative abundance of Firmicutes. At the class level, Bacteroidia and Clostridia were most abundant in several samples, with interventions such as F displaying consistent taxonomic profiles. At the order level, Clostridiales and Bacteroidales were prevalent in specific samples, while at the family level, Lactobacillaceae and Lachnospiraceae were more abundant in certain interventions like C1 and F2. The genus level clustering revealed that Lactobacillus, Bacteroides, and Muribaculum varied across interventions, with interventions F and G sharing similar distributions. Finally, species-level clustering highlighted individual species such as *Lactobacillus johnsonii* and *Muribaculum intestinale*, which exhibited intervention-dependent abundance. The heatmaps, normalized by z-scores, provided clear insights into microbial diversity, showcasing both shared and distinct microbial community profiles across intervention groups.

#### 3.2.5 Sample group distinction through PCA

**FIGURE 3:**
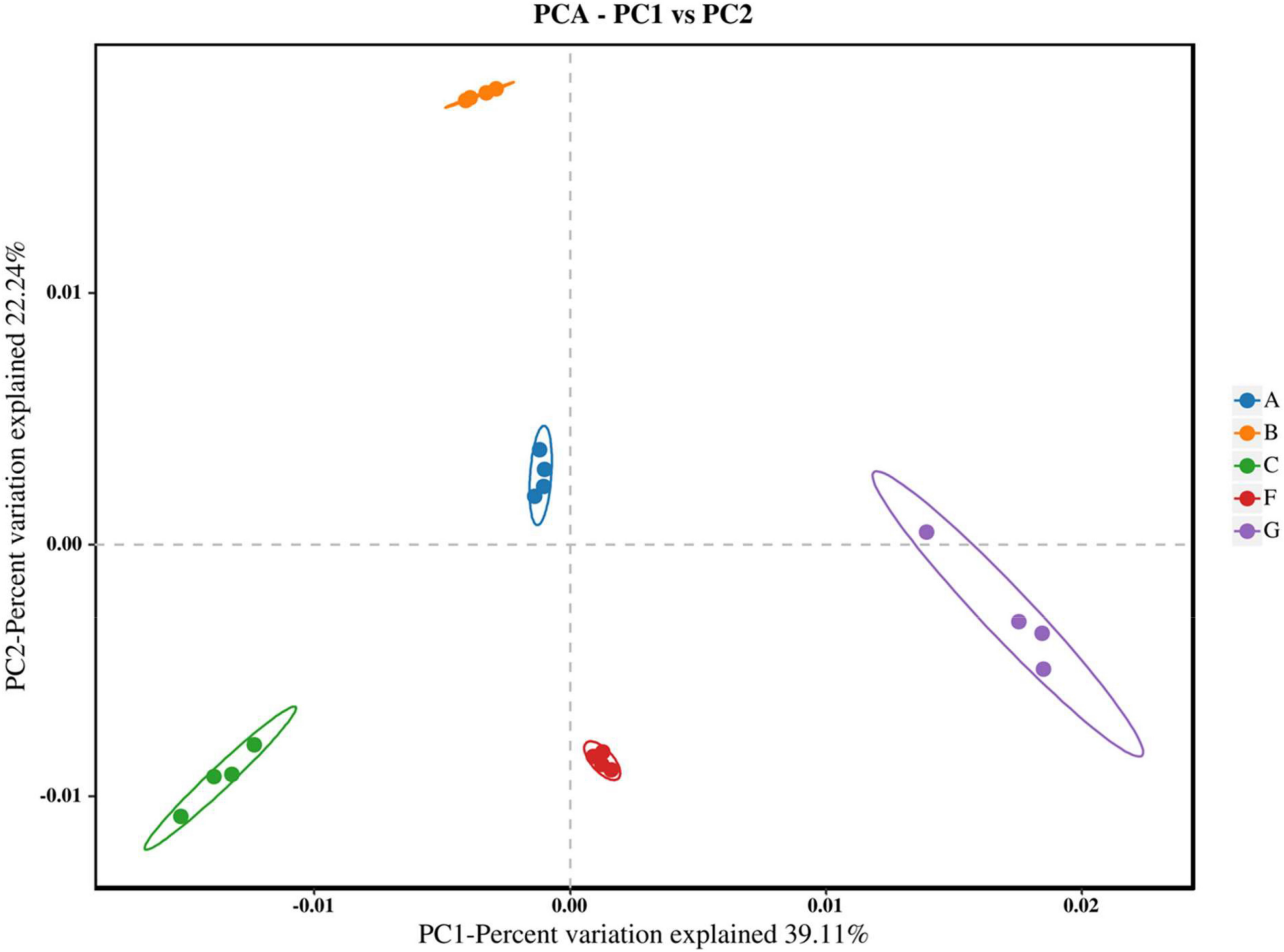
PCA analysis of samples from five intervention groups (Group A, Group B, Group C, Group F and Group G) (*n* = 4).

The PCA scatter plot, illustrates the distribution of samples along the first two principal components (PC1 and PC2) (Figure 3). The *X*-axis represents PC1, which accounts for 39.11% of the total variance in the dataset, while the *Y*-axis represents PC2, which explains 22.24% of the variance. The samples from different groups are well-separated in the plot, indicating significant differences in microbial composition between the interventions. For example, A and B groups are located in the upper-left region, while F and G are positioned in the lower-right quadrant. The ellipses suggest that the microbial communities within each intervention group are relatively cohesive but distinct from other groups. This PCA plot effectively summarizes the microbial composition variations between the intervention groups, providing insights into how different interventions influence the microbial community structure.

#### 3.2.6 Correlation patterns among microbial genera

**FIGURE 4:**
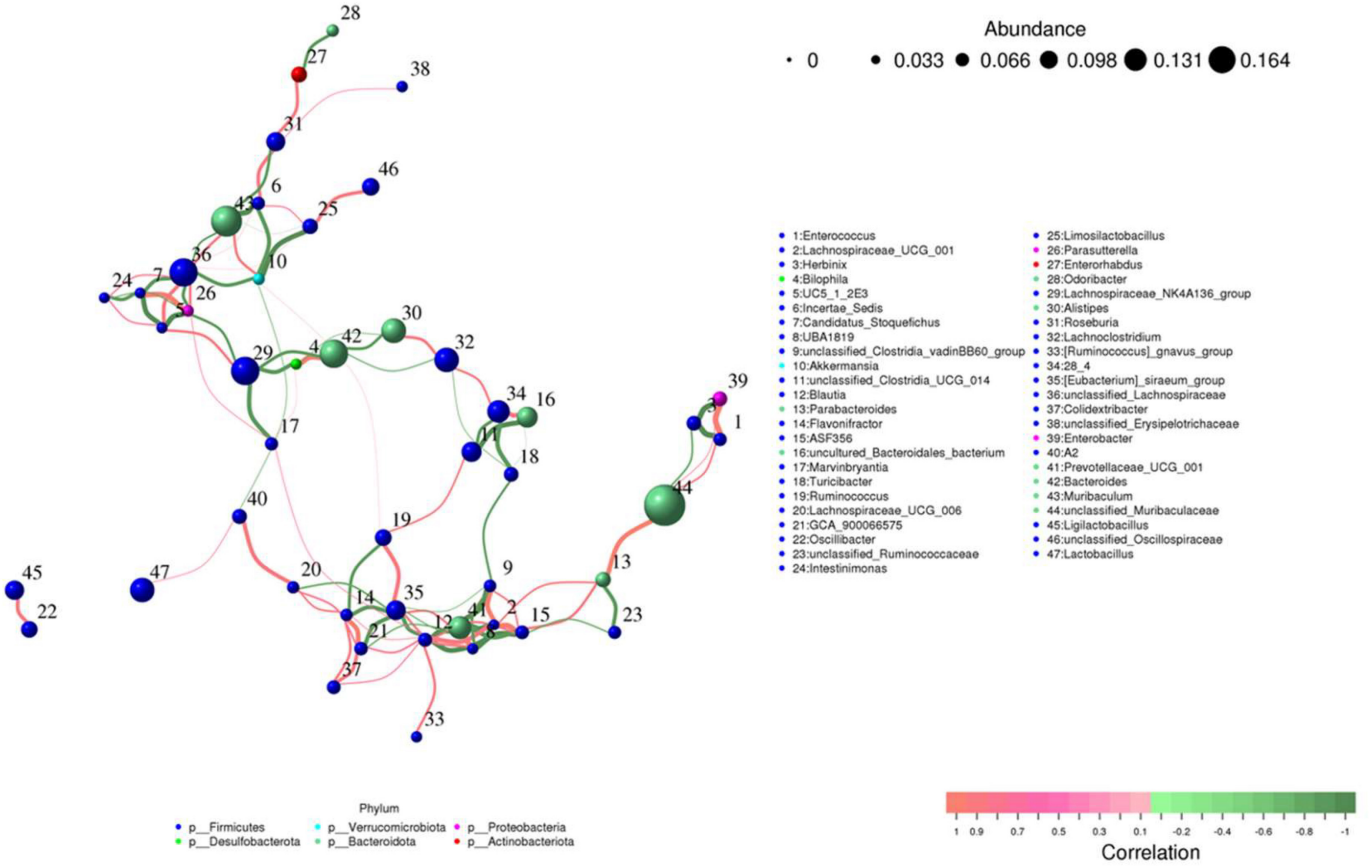
Species network at genus level.

A microbial co-occurrence network analysis was conducted to explore the relationships among different microbial taxa at the genus level (Figure 4). The network consists of nodes representing microbial genera, with node size proportional to the relative abundance of each genus. The color of each node corresponds to its taxonomic classification at the phylum level, including Firmicutes, Desulfobacterota, Verrucomicrobiota, Bacteroidota, Proteobacteria and Actinobacteriota. Several key taxa with high abundance were identified, such as genera 11 (unclassified Clostridia UCG_014, Firmicutes), 29 (Lachnospiraceae NK4A136 group, Firmicutes) and 41 (Prevotellaceae UCG_001, Bacteroidota), indicated by their larger node sizes. The network reveals complex interaction patterns within and between phyla, highlighting the potential ecological relationships that may contribute to the community structure.

The Firmicutes phylum dominated the network in terms of both abundance and connectivity, suggesting its central role in the microbial community. Genera within the Actinobacteriota and Proteobacteria phyla also showed specific positive and negative associations with other taxa, indicating potential niche specialization or competitive interactions.

#### 3.2.7 Clusters of orthologous groups (COG) functional profiling of gut microbiota in response to fermented sour soup intervention

**FIGURE 5:**
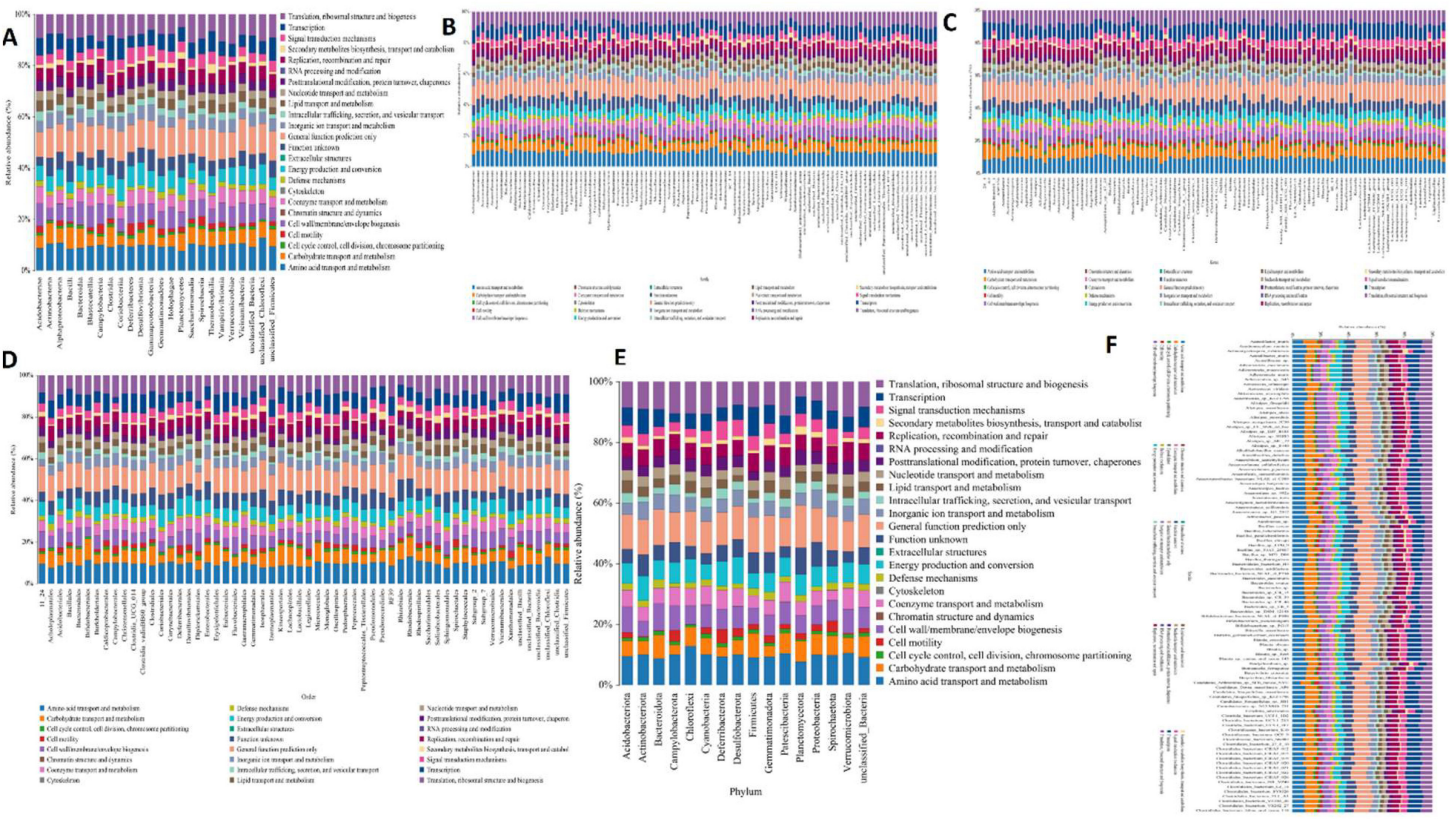
COG functional profiling of gut microbiota at taxonomic level: **(A)** Class; **(B)** Family; **(C)** Genus **(D)** Order; **(E)** Phylum; **(F)** Species.

Functional analysis of the gut microbiota was performed using COG to assess the impact of fermented sour soup on microbial activity at various taxonomic levels (Figures 5A–F). At the phylum level, prominent functional categories included amino acid and carbohydrate metabolism, energy production, and general function prediction. Firmicutes and Bacteroidota were most abundant in these functions, reflecting their roles in fermentation and nutrient processing. Proteobacteria exhibited functions linked to replication, recombination, and stress responses, suggesting adaptive microbial responses to dietary changes. Phyla such as Actinobacteriota and Verrucomicrobiota were enriched in lipid metabolism and cellular maintenance, while unclassified phyla contributed significantly to core metabolic functions, indicating functional importance despite limited taxonomic identification.

At the genus level, genera like *Bacteroides*, *Lactobacillus*, *Faecalibacterium*, and *Prevotella* were central to amino acid and carbohydrate metabolism, indicating their roles in nutrient processing and energy harvesting. Specific genera such as *Akkermansia*, *Alistipes*, and *Ruminococcus* were involved in lipid metabolism, secondary metabolite biosynthesis, and immune modulation. Some genera, including *Escherichia-Shigella* and *Klebsiella*, showed increased expression of genes related to stress responses and host defense mechanisms, potentially reflecting microbial adaptation to the fermented sour soup diet. Additionally, less-characterized genera like *Muribaculum* exhibited functions linked to unknown or general predictions, suggesting novel pathways that could play a role in host-microbe interactions.

At the order level, dominant functional categories such as amino acid metabolism, carbohydrate transport, and energy production were present in orders like Bacteroidales, Lachnospirales and Clostridiales. These orders play key roles in digestion and energy extraction, particularly from complex dietary substrates like fermented sour soup. Orders such as Enterobacterales and Desulfovibrionales showed functions related to replication, recombination, and defense mechanisms, indicating microbial activity in response to the dietary intervention. Some unclassified orders, such as RF39 and Peptococcales, still contributed to core metabolic functions, highlighting their potential functional relevance despite taxonomic uncertainty.

At the species level, core functional categories like amino acid transport, carbohydrate metabolism, and energy conversion were prevalent in species such as *Bacteroides thetaiotaomicron*, *Lactobacillus murinus*, and *Faecalibacterium prausnitzii*, which are known for their beneficial roles in gut health and nutrient metabolism. Species like *Akkermansia muciniphila* and *Bifidobacterium* contributed to lipid metabolism and cell wall biogenesis, underlining their involvement in gut mucosal integrity and energy homeostasis. Some less-characterized species, such as *Muribaculum intestinale*, exhibited disproportionately high levels of functions that are currently unknown, suggesting the presence of novel pathways related to the microbial adaptation to fermented sour soup. Species such as *Escherichia coli* and *Enterococcus faecalis* showed heightened levels of functions related to stress responses, genomic plasticity, and posttranslational modification, which may reflect active adaptation to the dietary intervention.

#### 3.2.8 COG functional category enrichment analysis of gut microbiota following fermented sour soup intervention: comparison between sample groups

**FIGURE 6:**
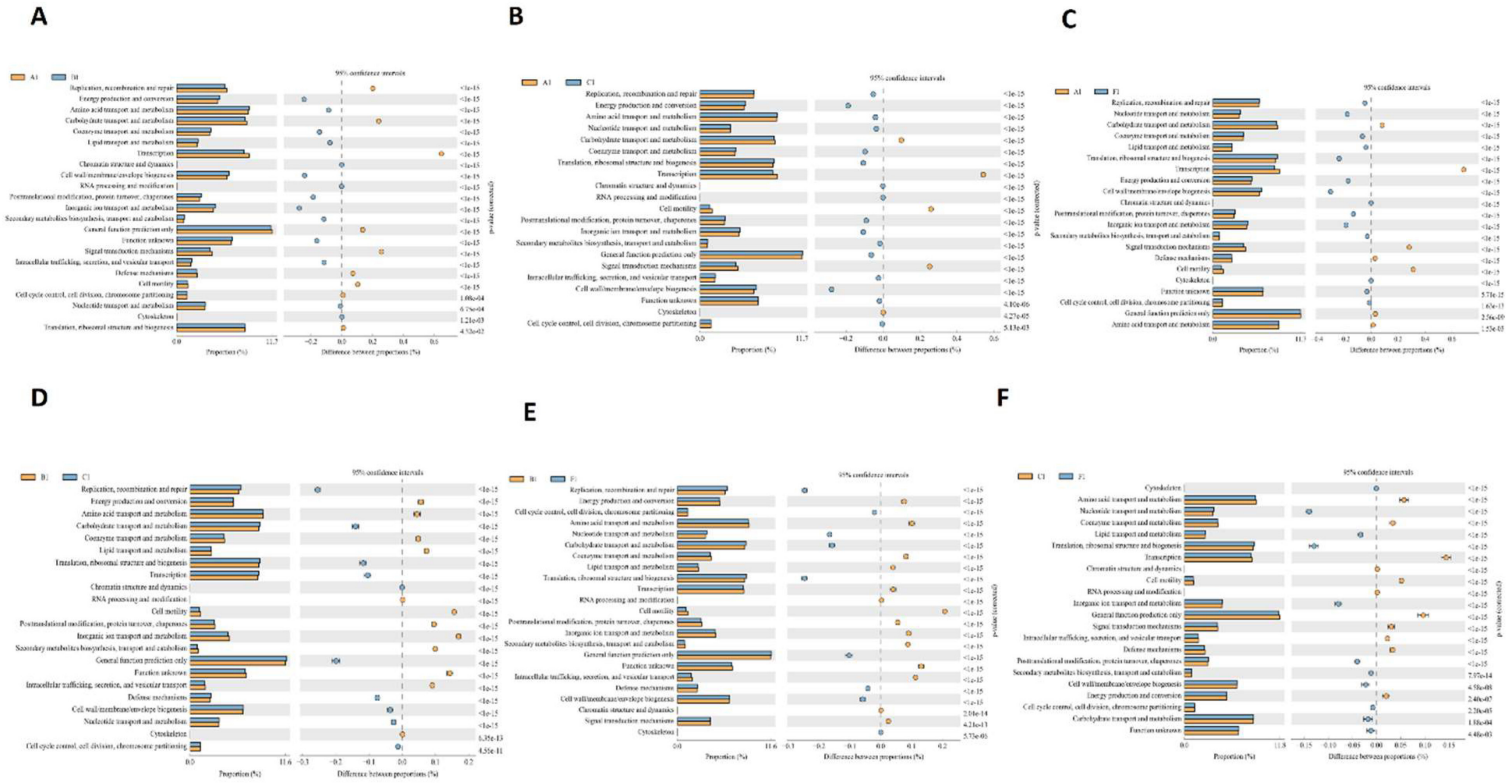
COG analysis of fecal samples revealed distinct functional differences in the gut microbiota. **(A)** Group A vs. group B; **(B)** group A vs. group C; **(C)** group A vs. group F; **(D)** group B vs. group C; **(E)** group B vs. group F; **(F)** group C vs. group F.

COG analysis of fecal samples revealed distinct functional differences in the gut microbiota between groups A, B, C, F, and G (Figures 6A–F). Group A exhibited significantly higher proportions of genes related to replication, recombination and repair, transcription, signal transduction, translation, ribosomal structure and biogenesis, cell motility, and defense mechanisms, indicating a microbial community with enhanced capabilities for genetic maintenance, protein synthesis, environmental responsiveness, and stress resistance. In contrast, group B showed enrichment in functions related to inorganic ion and amino acid transport and metabolism, secondary metabolite biosynthesis, energy production, cell wall/membrane biogenesis, and protein quality control, suggesting a microbiota more focused on metabolic activity and structural adaptation under stress. Similarly, group C shared several functional features with group B, including higher representation of genes involved in energy metabolism, membrane biogenesis, and secondary metabolite production, along with posttranslational modification and chaperone functions, highlighting a potential microbial capacity for resilience and bioactive compound synthesis. Across all groups, chromatin structure, RNA processing, and general function prediction remained relatively consistent, although subtle shifts in amino acid metabolism and gene regulation were noted. These findings suggest that fermented sour soup modulates gut microbiota function by promoting a more active and adaptive microbial community, particularly in groups A and C, while enhancing metabolic and structural support functions in group B.

Group A showed significantly higher proportions of genes related to cell motility, transcription, signal transduction mechanisms, and defense mechanisms, suggesting a microbial community with heightened environmental sensing, gene regulation, motility, and stress resistance indicative of a dynamic and adaptable microbiota. In contrast, group F was enriched in genes associated with inorganic ion transport and metabolism, cell wall/membrane/envelope biogenesis, secondary metabolite biosynthesis, nucleotide metabolism, posttranslational modification, energy production, and translation processes, reflecting enhanced microbial metabolic activity, structural maintenance, and protein quality control. While both groups exhibited high levels of transcription-related genes, the distribution slightly favored group A, highlighting its stronger regulatory potential. Subtle differences in signal transduction and posttranslational functions further suggest group-specific microbial strategies for adaptation and host interaction.

Group B showed significant enrichment in genes related to cell motility, secondary metabolite biosynthesis and transport, inorganic ion transport and metabolism, lipid metabolism, amino acid transport, posttranslational modification, and signal transduction, indicating a metabolically active microbiota with enhanced motility, metabolite production, and protein maintenance. In contrast, group C was characterized by higher proportions of genes involved in replication, recombination and repair, defense mechanisms, cell cycle control, translation, cell wall biogenesis, nucleotide metabolism, RNA processing, and amino acid transport, suggesting a microbiota with robust growth, genetic stability, and defense capabilities. Group F displayed enrichment in nucleotide transport and metabolism, translation, replication and repair, cell cycle control, carbohydrate metabolism, and cell wall biogenesis, reflecting a community oriented toward protein synthesis, genomic maintenance, and structural functions.

#### 3.2.9 KEGG functional annotation of gut microbiota

**FIGURE 7:**
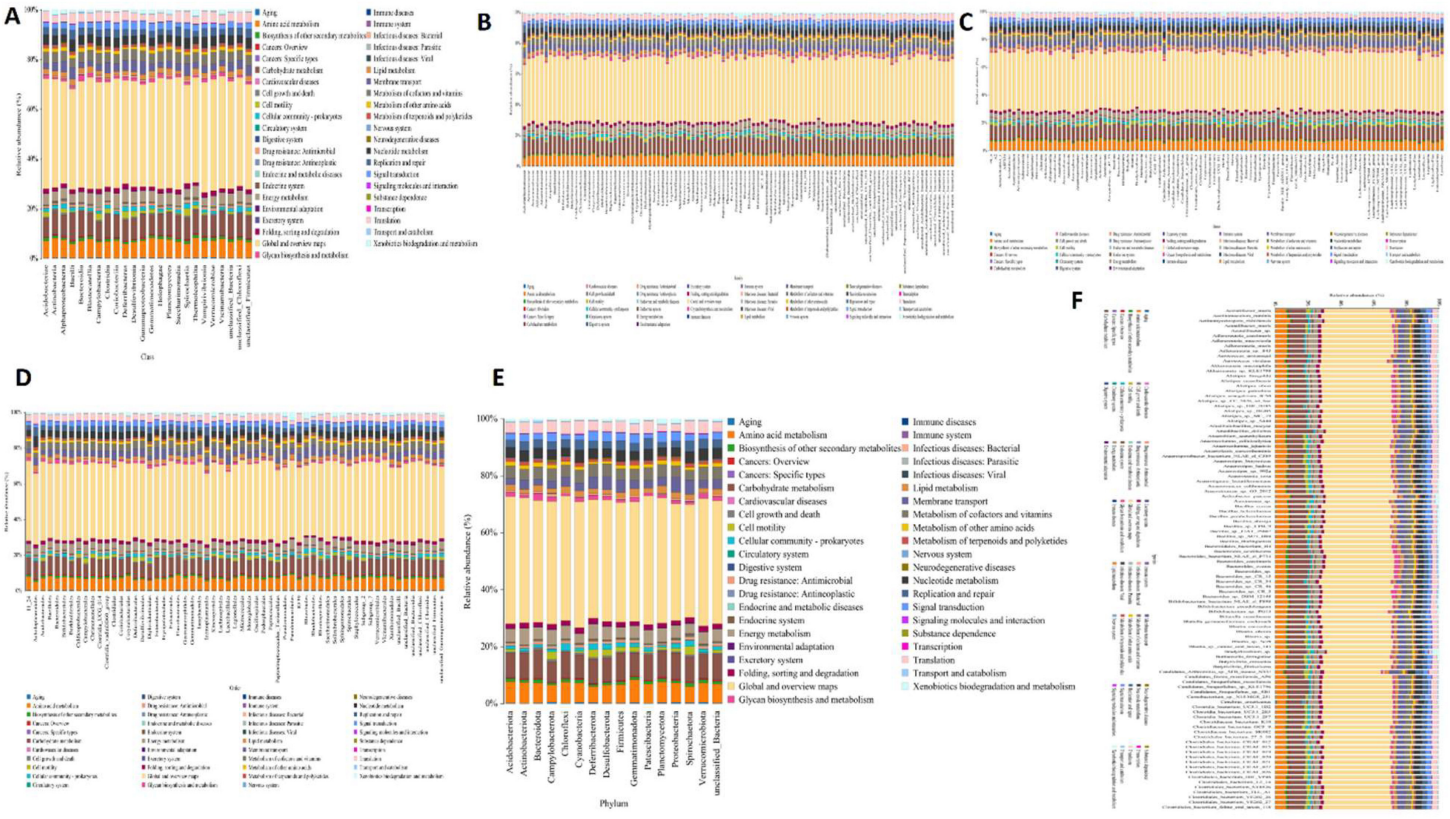
Functional annotation of gut microbiota at the taxonomic level. **(A)** Class; **(B)** Family; **(C)** Genus **(D)** Order; **(E)** Phylum; **(F)** Species (*p* < 0.05).

KEGG pathway analysis of gut microbiota from mice treated with fermented sour soup revealed a consistent and comprehensive functional profile across multiple taxonomic levels, highlighting extensive metabolic and adaptive microbial shifts associated with the dietary intervention. Across bacterial classes, the dominant functional category was “Global and overview maps,” which accounted for a substantial portion of the relative abundance, particularly in taxa such as Bacteroidia, Clostridia, Gammaproteobacteria, and Bacilli (Figure 7A). This finding underscores the broad representation of essential core metabolic functions, including central carbon metabolism and biosynthesis of vital compounds. Key pathways related to carbohydrate metabolism, amino acid metabolism, and energy metabolism were consistently enriched, especially in classes like Clostridia, Bacteroidia, and Bacilli, indicating active participation in nutrient breakdown, fermentation, and energy extraction processes that are integral to host detoxification and maintenance of gut-liver axis health. Lipid metabolism, membrane transport, and xenobiotics biodegradation and metabolism pathways were notably prominent in Actinobacteria, Gammaproteobacteria, and Verrucomicrobiae, suggesting microbial involvement in bile acid transformation, detoxification of harmful substances, and degradation of fermentation by-products or alcohol-derived compounds. Functional categories associated with genetic information processing, including replication and repair, transcription, translation, and folding/sorting/degradation, were prevalent across classes, reflecting a dynamic and adaptable microbial community responding to fermented sour soup intake. Moderate representation of environmental adaptation, signal transduction, and cell motility in classes such as Campylobacteria, Spirochaetia, and Desulfovibrionia further indicated microbial responsiveness to ecological shifts within the gut environment. Health-related pathways, including those related to infectious diseases, drug resistance, and endocrine and metabolic diseases, were detected at relatively low abundances, suggesting a stable and less pathogenic microbial composition after intervention.

At the family level, “Global and overview maps” consistently dominated, with families such as Lachnospiraceae, Ruminococcaceae, Bacteroidaceae, and Prevotellaceae exhibiting a shared foundation of core metabolic and biosynthetic functions crucial for microbial-host metabolic coordination (Figure 7B). Carbohydrate, amino acid, and energy metabolism pathways were highly prevalent in families including Lactobacillaceae, Muribaculaceae, Erysipelotrichaceae, and Oscillospiraceae, reflecting enhanced fermentation, energy harvesting, and metabolite production following sour soup consumption. Families such as Enterobacteriaceae, Desulfovibrionaceae, and Coriobacteriaceae showed enrichment in lipid metabolism, membrane transport, and xenobiotics biodegradation, implying roles in detoxification, fermentation by-product absorption, and lipid metabolism modulation relevant to liver protection. Genetic information processing pathways were evenly distributed, supporting active microbial growth and adaptation, while lower abundance categories such as signal transduction, cell motility, environmental adaptation, and drug resistance appeared in families like Peptostreptococcaceae and Helicobacteraceae, indicating microbial communication and survival strategies within the gut ecosystem.

At the genus level, “Global and overview maps” remained predominant, particularly enriched in genera such as Lactobacillus, Bacteroides, Alistipes, Ruminococcus, and Faecalibacterium, which play key roles in central metabolism and systemic microbial-host interactions (Figure 7C). Core metabolic pathways were widespread, with carbohydrate, amino acid, and energy metabolism enriched in Bacteroides, Lactobacillus, and Muribaculum, supporting fermentation and biosynthesis of beneficial metabolites involved in detoxification and hepatoprotection. Genera including Escherichia, Blautia, Desulfovibrio, and Clostridium showed increased involvement in lipid metabolism, membrane transport, and xenobiotics degradation, highlighting their contribution to bile acid modulation and alcohol-derived compound breakdown under fermented food intervention. Genetic information processing pathways were universally present, signifying a dynamic microbial environment adapting to dietary influences. Additionally, signal transduction, environmental adaptation, and drug resistance pathways were found in Helicobacter, Peptostreptococcus, and Enterococcus, reflecting microbial stress responses likely influenced by sour soup bioactive compounds. Low-abundance pathways linked to infectious diseases, immune function, and endocrine regulation suggested a generally balanced and non-pathogenic microbiota profile post-intervention.

Analysis at the order level confirmed the predominance of “Global and overview maps” in groups such as Lactobacillales, Bacteroidales, Clostridiales, Erysipelotrichales, and Muribaculales, reinforcing the conserved nature of core metabolic functions across gut taxa (Figure 7D). Carbohydrate, amino acid, and energy metabolism were highly enriched in Lactobacillales, Bacteroidales, and Oscillospirales, consistent with roles in dietary component fermentation and bioactive metabolite production supporting liver-protective effects. Orders like Desulfovibrionales, Enterobacterales, and Coriobacteriales showed elevated lipid metabolism, membrane transport, and xenobiotic degradation, suggesting involvement in host detoxification and gut-liver metabolic signaling post-alcohol exposure. Replication, repair, transcription, translation, and protein folding pathways were widespread, indicating active microbial adaptation. Low-level detection of pathways related to infectious diseases, immune modulation, and neurodegeneration in orders such as Clostridiales and Enterobacterales provided insight into microbial influences on host systemic responses. Niche functions like environmental adaptation, signal transduction, drug resistance, and cell motility distributed among Desulfovibrionales and Spirochaetales further illustrated microbial plasticity in the sour soup-modulated gut environment.

Phylum-level analysis mirrored these patterns, with “Global and overview maps” dominating among Firmicutes, Bacteroidota, Actinobacteriota, Proteobacteria, Verrucomicrobiota, and Cyanobacteria, highlighting the conserved presence of broad metabolic and cellular processes (Figure 7E). Carbohydrate, amino acid, and energy metabolism were enriched, reflecting the microbiota’s active role in nutrient breakdown and energy production critical for liver function and detoxification. Firmicutes and Bacteroidota showed higher gene abundances related to glycan biosynthesis, folding and degradation, and membrane transport, indicating roles in complex carbohydrate digestion and metabolite exchange. Genetic information processing functions were consistent across phyla, evidencing active microbial growth and adaptation. Lower but consistent levels of pathways related to infectious diseases, immune system, and drug resistance were noted in Proteobacteria and Actinobacteriota, suggesting microbial contributions to host immunity and xenobiotic metabolism. Verrucomicrobiota and Cyanobacteria displayed unique enrichment in xenobiotics biodegradation, emphasizing their detoxification roles.

At the species level, the “Global and overview maps” category prevailed across diverse bacteria, highlighting general metabolic and cellular processes fundamental for microbial viability and host interaction (Figure 7F). Carbohydrate, amino acid, and energy metabolism pathways were significantly enriched, supporting nutrient processing and energy production essential for host metabolic homeostasis and liver protection. Certain species showed high gene abundance in glycan biosynthesis, folding, sorting, degradation, and membrane transport, reflecting roles in carbohydrate digestion, protein quality control, and metabolite exchange. Replication, repair, transcription, and translation pathways were widespread, indicating active microbial growth and gene expression dynamics driven by fermented sour soup intervention. Low-level presence of pathways related to infectious diseases, immune system, and drug resistance, particularly in opportunistic taxa, suggested modulation of microbial contributions to host immunity and resilience. Xenobiotics biodegradation pathways in select species implied their involvement in detoxification and metabolite transformation supporting liver health.

#### 3.2.10 KEGG pathway enrichment analysis of gut microbiota post fermented sour soup intervention: comparison between groups

**FIGURE 8:**
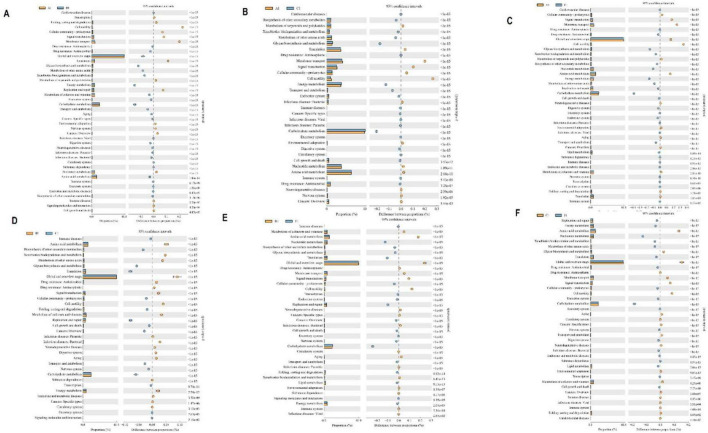
Analysis of KEGG metabolic pathways difference between groups. **(A)** Group A vs. group B; **(B)** group A vs. group C; **(C)** group A vs. group F; **(D)** group B vs. group C; **(E)** group B vs. group F; **(F)** group C vs. group F (*p* < 0.05).

Comparative KEGG pathway analysis between experimental groups revealed distinct shifts in microbial functional profiles associated with fermented sour soup intervention. Group B showed significant enrichment in infectious disease-related pathways (parasitic, viral, bacterial), immune system, and circulatory system functions compared to group A, indicating enhanced microbial capacities for pathogen response and immune modulation (*p*-values < 4e^–05^) (Figure 8A). Conversely, group A exhibited higher representation of pathways involved in replication and repair, translation, and neurodegenerative diseases, suggesting more active microbial maintenance and protein synthesis under control conditions. Metabolic pathways such as carbohydrate and amino acid metabolism were significantly upregulated in group B (*p* < 0.002), reflecting increased nutrient processing and energy extraction following intervention, whereas group A showed elevated pathways related to cell motility and microbial community interactions. Comparison of group A and C revealed that group C had greater enrichment in carbohydrate metabolism, energy metabolism, and glycan biosynthesis pathways (*p* < 0.0005), suggesting enhanced microbial energy production and host interaction, while group A had higher levels of cell motility, signal transduction, and translation, highlighting a community with greater environmental sensing and communication (Figure 8B). Group C also demonstrated increased biosynthesis of secondary metabolites and terpenoid metabolism, contrasting with elevated nervous system of group A and signal transduction functions.

In the A vs. F comparison, group F showed significant enrichment in carbohydrate, energy, and lipid metabolism pathways (*p* < 3e^–04^), alongside transport, catabolism, and antimicrobial drug resistance pathways, suggesting improved microbial adaptability and resilience (Figure 8C). Group A had higher abundances in cell motility, signal transduction, amino acid metabolism, and neurodegenerative disease-related pathways, indicating a different microbial functional emphasis. The B versus C comparison highlighted that group C exhibited stronger capacities for replication, repair, translation, transcription, and glycan metabolism (*p* < 0.001), supporting enhanced microbial growth and host glycan utilization, whereas group B showed elevated signal transduction, cell motility, xenobiotic metabolism, and amino acid metabolism, indicative of a more dynamic and metabolically versatile microbial community (Figure 8D). Both groups differed in infectious disease and neurodegenerative disease pathways, with group C generally showing reduced disease-associated functions. Analysis between groups A and F demonstrated that group F had higher levels of replication and repair, transcription, translation, carbohydrate and nucleotide metabolism, and lipid metabolism pathways (*p* < 5e^–05^), consistent with greater microbial growth, energy production, and detoxification potential (Figure 8E), while group B exhibited increased signal transduction, amino acid metabolism, cell motility, aging, neurodegenerative disease, and infectious disease pathways, suggesting a microbial profile associated with host stress or disease susceptibility. Finally, comparison of groups C and F revealed group F’s microbiota had most probably enhanced xenobiotic biodegradation, nucleotide, carbohydrate, and lipid metabolism (*p* < 2e^–04^), as well as digestive system and replication functions, indicating stronger detoxification and metabolic activity, whereas group C showed elevated neurodegenerative disease, aging, infectious diseases, amino acid metabolism, signal transduction, cell motility, immune system, and cancer-related pathways, reflecting a microbial community potentially linked to inflammatory or pathological states (Figure 8F).

#### 3.2.11 Predicted phenotypic traits of the microbiome reveal functional divergence across sample groups

**FIGURE 9:**
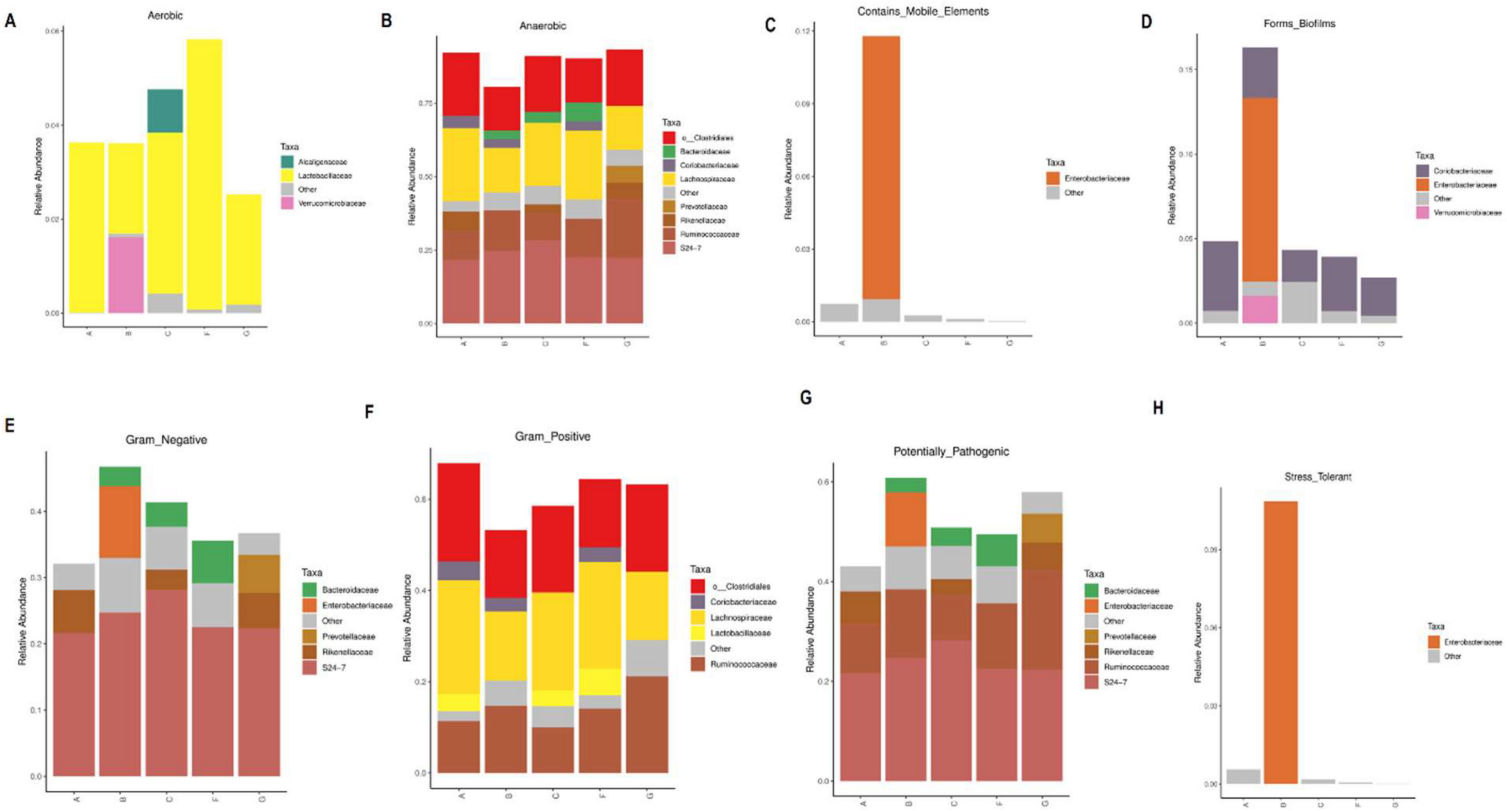
Functional and phenotypic traits of gut microbial taxa across sample groups. **(A)** Aerobic; **(B)** Anaerobic; **(C)** Contains_mobile elements; **(D)** Forms_biofilms; **(E)** Gram-negative; **(F)** Gram-positive; **(G)** Potentially_pathogenic; **(H)** Stress_tolerant.

To assess the variation in microbial composition across different sample groups (A, B, C, F, and G), the relative abundances of microorganisms were analyzed. As shown in Figure 9A, the relative abundance of aerobic taxa differed significantly among the groups. Group F exhibited the highest relative abundance of aerobic microbes (mean ∼0.063), followed by group C (∼0.050), group B (∼0.036), and group A (∼0.037). The lowest abundance was observed in group G (∼0.026). These results indicate a heterogeneous distribution of aerobic taxa, with certain groups (particularly F and C) showing elevated levels of aerobic bacteria, potentially reflecting differences in environmental oxygen availability or host-related factors.

In contrast, Figure 9B illustrates that anaerobic microorganisms constituted the vast majority of the microbial community across all groups, with relative abundances exceeding 80% in every case. Group G demonstrated the highest anaerobic abundance (mean ∼0.938), followed closely by group A (∼0.922), group C (∼0.914), and group F (∼0.904). Group B showed a relatively lower proportion of anaerobes (∼0.808), aligning with its slightly elevated aerobic abundance. The dominance of anaerobic taxa across groups is consistent with the expected microbial ecology of anaerobic environments, such as the gastrointestinal tract.

For the “Contains_Mobile_Elements” phenotype, organisms belonging to group B exhibited a markedly higher relative abundance compared to all other groups, with values ranging between 0.11 and 0.14 (Figure 9C). In contrast, groups A, C, F, and G displayed minimal abundance levels close to zero, suggesting that mobile genetic elements were largely restricted to members of group B. This finding indicates a greater potential for horizontal gene transfer and genomic plasticity within group B organisms. Analysis of the “Forms_Biofilms” phenotype showed a similar trend (Figure 9D). Group B organisms demonstrated the highest predicted biofilm-forming potential, with relative abundance values between 0.15 and 0.18. Other groups exhibited consistently lower abundances, with group A showing modest levels (∼0.05), while groups C, F, and G remained below 0.05. These results suggest that biofilm-associated traits are enriched in group B, which may enhance persistence, colonization, and antimicrobial resistance within this community. The “Gram_Negative” phenotype prediction demonstrated broader distribution across groups (Figure 9E). Group B again exhibited the highest abundance (0.46–0.48), followed by group C (∼0.41–0.42), while groups F and G displayed moderate levels (0.35–0.37). Group A showed the lowest proportion of Gram-negative organisms (0.31–0.33). The enrichment of Gram-negative phenotypes in groups B and C highlights the dominance of bacteria with outer membrane-associated features, which are often linked to pathogenicity and resistance mechanisms. Across all groups, Lachnospiraceae and Ruminococcaceae were the dominant families, with Lachnospiraceae representing the highest proportion of Gram-positive bacteria, particularly in groups A and F (Figure 9F). Ruminococcaceae maintained a relatively stable abundance across all groups, contributing approximately 20–25% of the Gram-positive community. Group A showed the highest overall relative abundance of Gram-positive bacteria, largely driven by elevated levels of Lachnospiraceae, Lactobacillaceae, and taxa classified under the order Clostridiales. Conversely, group B exhibited the lowest relative abundance of Gram-positive bacteria, with decreased levels of Clostridiales and Lactobacillaceae. Group G showed a notable increase in the proportion of “Other” Gram-positive taxa and Ruminococcaceae, suggesting a shift in the Gram-positive community structure compared to other groups. The presence of Coriobacteriaceae was minor but detectable in groups A, B, and F. The relative abundance of potentially pathogenic microbes varied significantly across groups (Figure 9G). Group B exhibited the highest abundance (mean ≈ 0.61), followed by group G (≈ 0.58), indicating a higher predicted pathogenic potential in these groups. In contrast, group A showed the lowest abundance of potentially pathogenic organisms (≈ 0.43), suggesting a lower risk of pathobiont expansion or opportunistic infection. Groups C and F exhibited intermediate levels (∼0.51 and ∼0.49, respectively), highlighting a gradient of potential pathogenicity that may be reflective of underlying environmental or host-associated differences. Predictions for stress-tolerant phenotypes demonstrated even starker contrasts among groups (Figure 9H). Group B again stood out with a markedly elevated abundance of stress-tolerant taxa (mean ≈ 0.11), suggesting a microbial community that may be adapting to challenging or perturbed environments. All other groups (A, C, F, and G) exhibited extremely low levels of stress-tolerant organisms (close to 0.01 or lower), indicative of more stable or less selective microbial habitats.

As shown in Figure 9B, the anaerobic phenotype was predominant across all samples, with the relative abundance consistently high, especially in samples A, C, F, and G. The most dominant taxa associated with anaerobic function were S24–7, Ruminococcaceae and Lachnospiraceae, which together accounted for the majority of the microbial community composition. The order Clostridiales was a major contributor across all samples, particularly dominant in samples A and G. Sample B exhibited a relatively lower total abundance of anaerobic taxa, suggesting a possible variation in anaerobic potential among subjects. Predictions for the “Contains Mobile Elements” phenotype (Figure 9C) revealed a striking enrichment of Enterobacteriaceae in sample B, where it accounted for nearly the entire mobile element-associated microbial fraction. Other samples exhibited minimal contributions from this phenotype. This suggests that sample B may harbor a microbial community with a higher potential for horizontal gene transfer, which could be associated with pathogenicity or antibiotic resistance. The “Forms Biofilms” phenotype (Figure 9D) demonstrated a similar trend, with Enterobacteriaceae again dominating sample B. This group was nearly absent or minimally present in other samples. Coriobacteriaceae were consistently observed across all samples, suggesting a broader distribution of this taxon’s biofilm-forming capability. Sample B also exhibited unique contributions from Verrucomicrobiaceae, a taxon not detected in this phenotype in other samples. The presence of Enterobacteriaceae as the dominant biofilm-forming taxon in sample B underscores its potential role in host-associated pathophysiological processes.

The relative abundance of Gram-negative bacterial taxa varied among the five sample groups (A, B, C, F, and G) (Figure 9E). The dominant taxon across all samples was S24-7, representing approximately 22–30% of the total Gram-negative community. Sample B showed the highest overall abundance of Gram-negative bacteria, driven largely by a notable increase in Enterobacteriaceae (∼13%), which was not detected at similar levels in the other samples. Bacteroidaceae was also more abundant in samples F and B compared to others. Minor contributions from Prevotellaceae and Rikenellaceae were observed across samples, with some variation, while the “Other” taxa accounted for approximately 5–10% of the community. The Gram-positive bacterial community showed distinct taxonomic profiles compared to Gram-negative bacteria (Figure 9F). The most abundant taxa across all samples were Lachnospiraceae and o__Clostridiales, each contributing between 20 and 30% of the community. Notably, sample A exhibited the highest total abundance of Gram-positive taxa, with a dominant presence of o__Clostridiales (∼25%). Ruminococcaceae was also consistently abundant across all samples (∼15–20%). Lactobacillaceae and Coriobacteriaceae contributed to a smaller extent, with Lactobacillaceae being more prevalent in samples A and F.

#### 3.2.12 Overview of ecological functional prediction and taxonomic comparisons across groups

**FIGURE 10:**
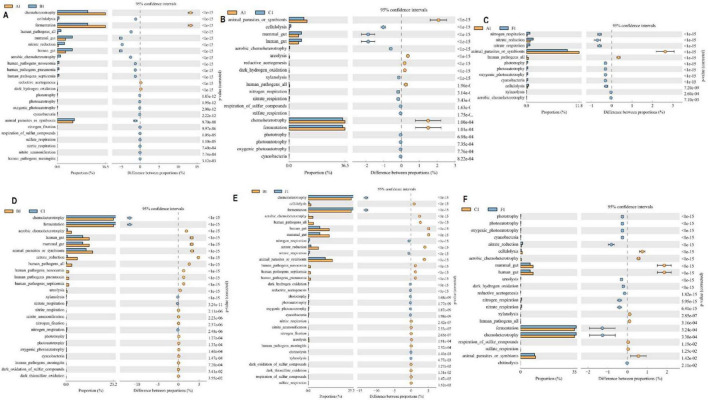
Overview of ecological functional prediction based on differential abundance. **(A)** Group A vs. group B; **(B)** group A vs. group C; **(C)** group A vs. group F; **(D)** group B vs. group C; **(E)** group B vs. group F; **(F)** group C vs. group F. (*p* < 0.05).

To assess functional differences across microbial communities, the relative proportions of annotated metabolic and taxonomic features between group A and three comparative groups: B, C and F were compared. The bar plots display proportions of each feature in both groups, while the forest plots represent the differences in proportions along with 95% confidence intervals and corrected *p*-values. Comparison between A and B (Figure 10A) revealed Fermentation and chemoheterotrophy were significantly enriched in group A compared to B, with a notable increase in fermentation. Conversely, functions such as human_pathogens_all, mammal_gut, nitrate_reduction and human_gut were significantly more abundant in B. Other enriched functions in B1 included multiple pathogen-associated terms (such as *nosocomia*, *pneumonia*, *septicemia*), alongside a strong presence of dark_hydrogen_oxidation, reductive_acetogenesis and phototrophy-related features.

In the A vs. C comparison (Figure 10B), the most pronounced differences were observed in animal_parasites_or_symbionts, mammal_gut and human_gut, all significantly enriched in C. Functions associated with ureolysis, xylanolysis, dark_hydrogen_oxidation and reductive_acetogenesis also showed higher proportions in C. In contrast, A was significantly enriched in fermentation, chemoheterotrophy, and human_pathogens_all, with marginal differences also observed for phototrophy, photoautotrophy, and oxygenic_photoautotrophy. Notably, several sulfur- and nitrogen-related respiratory pathways (such as nitrate respiration, sulfate respiration, respiration of sulfur compounds) were enriched in C. The A vs. F comparison (Figure 10C) highlighted strong enrichment of animal_parasites_or_symbionts in A compared to F. In contrast, F showed significantly higher levels of nitrogen respiration, nitrate reduction, and nitrate respiration, suggesting an enrichment of nitrogen cycling pathways. Additional functions including phototrophy, photoautotrophy, oxygenic_photoautotrophy and cyanobacteria were also more prevalent in F, although at lower absolute proportions. Cellulolytic functions and xylanolysis showed modest enrichment in F, while human_pathogens_all remained significantly more abundant in A. Across all comparisons, group A was consistently enriched in fermentation, chemoheterotrophy and pathogen-associated functions, while groups B, C, and F exhibited higher relative abundance of gut-associated, nitrogen/sulfur respiratory and phototrophic pathways. The comparison between groups B and C revealed significant differences in multiple microbial ecological functions (Figure 10D). Chemoheterotrophy, fermentation and aerobic chemoheterotrophy were significantly more abundant in both groups, but particularly enriched in C. Functions associated with host-associated environments, such as human gut, mammal gut, and animal parasites or symbionts, were more enriched in B, suggesting a higher representation of host-associated microbes in this group. Several pathogenic functional annotations, including human pathogens (all), nosocomial, pneumonia, and septicemia, were significantly more abundant in B compared to C. Conversely, environmental functions like ureolysis, nitrate respiration, nitrite respiration, and nitrogen fixation were enriched in C, suggesting a stronger representation of nitrogen cycling pathways in that group.

In the B vs. F comparison, significant functional differences were again observed across numerous traits (Figure 10E). Group B showed higher abundances in chemoheterotrophy, fermentation, and aerobic chemoheterotrophy, while F had a notably higher abundance in cellulolysis. Host-related functions (human gut, mammal gut, and animal parasites or symbionts) were significantly enriched in B, consistent with the B vs. C comparison. Environmental and autotrophic functions were more prevalent in F. Specifically, phototrophy, photoautotrophy, oxygenic photoautotrophy, and cyanobacteria were significantly enriched, indicating a higher representation of photosynthetic or phototrophic microorganisms. Functions involved in dark hydrogen oxidation, nitrogen respiration, and nitrate respiration were also more abundant in F, further highlighting its environmental/soil-associated functional profile. When comparing C1 and F1, a clear functional separation was observed, particularly in functions associated with phototrophy and nitrogen cycling (Figure 10F). The F group showed significantly higher abundances of phototrophy, photoautotrophy, oxygenic photoautotrophy and cyanobacteria, confirming the increased prevalence of photosynthetic microbes in this group. Conversely, C was enriched in host-associated functions such as mammal gut, human gut, and aerobic chemoheterotrophy, similar to the pattern observed in B. Additionally, fermentation and chemoheterotrophy were significantly more abundant in C. Functions such as ureolysis, reductive acetogenesis and dark hydrogen oxidation were significantly elevated in F, indicating metabolic specialization toward anaerobic and hydrogen-based metabolisms. Sulfur metabolism-related functions like respiration of sulfur compounds and sulfate respiration were significantly more enriched in F, suggesting the presence of sulfur-reducing microbial communities.

### 3.3 Metabolomics analysis results

#### 3.3.1 Principal component analysis (PCA) component analysis of sample groups

**FIGURE 11:**
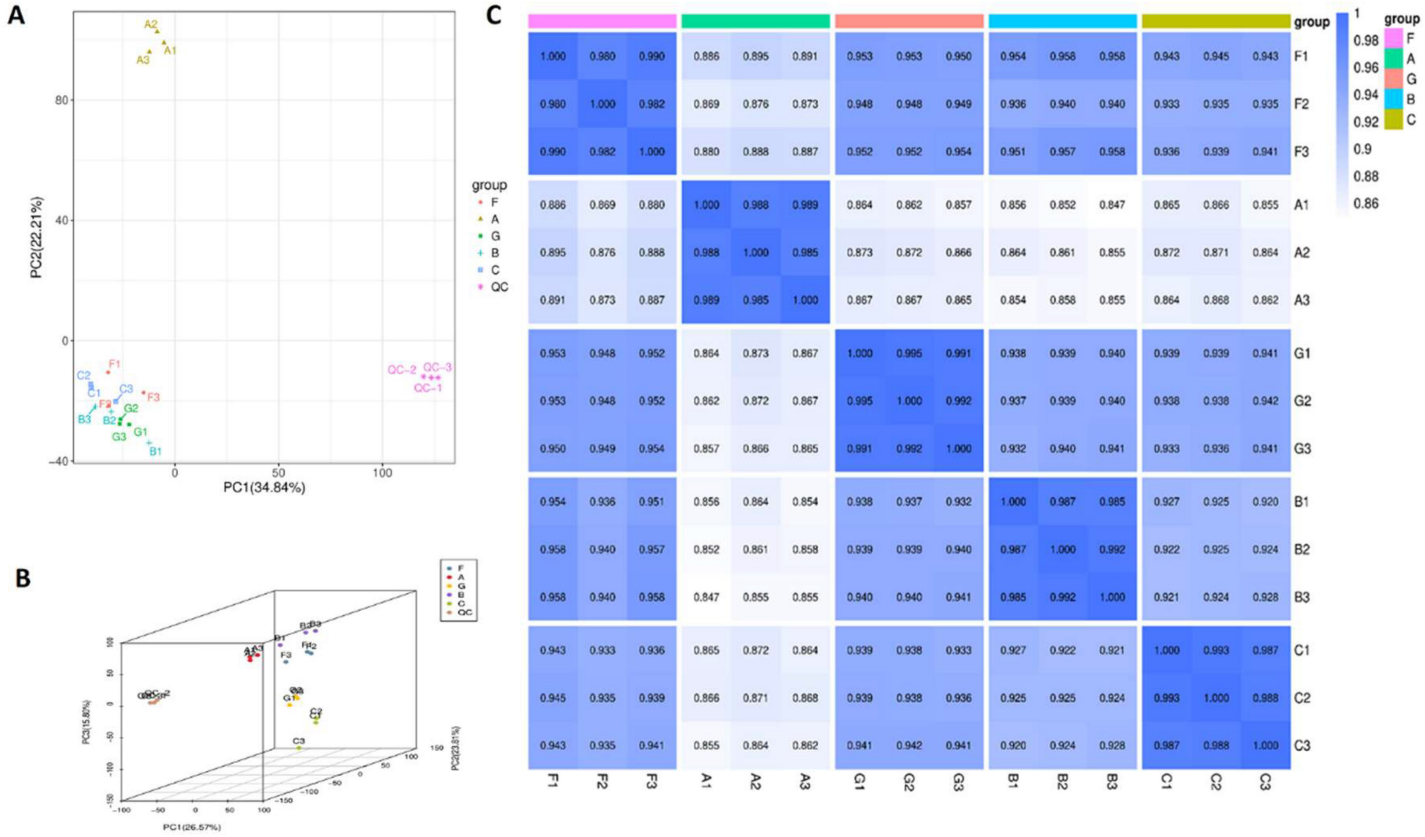
Metabolic profiling of fecal samples. **(A)** PCA score plot of sample groups. **(B)** PCA analysis of fecal metabolites. **(C)** Pearson correlation heat map of fecal metabolites (*n* = 3).

To evaluate the overall variation and clustering patterns among different sample groups, a PCA was conducted. The PCA score plot (Figure 11A) illustrates the distribution of six experimental groups: group A (blank group), group B (model group), group C (positive control group), group F (low dose intervention group), group G (high dose intervention group) and group QC (quality control group), based on the first two principal components, PC1 and PC2, which explain 34.84 and 22.21% of the total variance, respectively.

Group A (blank group) samples clustered tightly in the upper-left quadrant with high PC2 values, indicating a distinct separation from all other groups. In contrast, group B (model group) showed a marked shift along PC1 and PC2, indicating substantial metabolic or molecular changes induced by the model intervention. Group C (positive control) clustered closely with group B, but showed a slight trend toward group F and G, suggesting partial correction or modulation. Groups F (low dose) and G (high dose) were positioned between the model group (B) and blank group (A), with group G (high dose) samples clustering slightly closer to the blank group. This indicates a dose-dependent response and suggests that the intervention may partially reverse the model-induced alterations. The QC samples were clearly separated from all other groups and formed a tight cluster on the far right of the plot, reflecting high technical reproducibility and data stability.

#### 3.3.2 PCA analysis of fecal metabolites

To explore the metabolic changes associated with the consumption of fermented sour soup and its potential hepatoprotective effects, PCA was conducted on fecal metabolite profiles collected from mice (Figure 11B). Group A (samples A1-A3), representing the blank group, exhibited strong separation along the negative axis of PC1, with values ranging from -145.19 to -134.50. In contrast, Group B (B1–B3), which is a model group, clustered tightly in the positive PC1 and high PC2 quadrant (PC1 ∼44–46, PC2 ∼34–85), suggesting distinct metabolic profiles associated with the intervention. Group C (C1-C3), potentially representing a positive group, was positioned primarily in the negative PC2 region (PC2 ∼ -45 to -110), indicating a different metabolomics signature compared to both the control and intervention groups. Groups F and G, representing other experimental conditions such as intervention combinations or different dosage levels exhibited overlapping but distinguishable clustering in the positive PC1 range, with Group F (F1-F3) spread across PC2 values of 25–62, and Group G (G1-G3) clustering in the negative PC2 range (-40 to -60). The separation between groups suggests that the hepatoprotective effects may be mediated through distinct metabolic pathways.

#### 3.3.3 Correlation analysis of fecal metabolites

To further explore the relationships among metabolite profiles across different experimental groups, a Pearson correlation heatmap was generated (Figure 11C). Samples within the same group showed strong intra-group correlations, indicating high consistency in metabolic responses within interventions. For instance, Group F (F1-F3), representing low-dose mixed bacteria fermented sour soup group, exhibited very strong internal correlations (*r* = 0.98–0.99), suggesting homogeneity in metabolite expression. Similarly, Group A (A1-A3, blank group) also demonstrated high intra-group correlation coefficients (*r* = 0.88–0.89), indicating stable metabolic states in untreated mice. Group B (B1-B3), which is a model group, also showed excellent intra-group correlation (*r* = 0.99–1.00), reinforcing the reproducibility of the intervention effect. The positive group, Group C (C1-C3), revealed slightly lower but still strong correlations among its replicates (*r* = 0.98–0.99), suggesting a consistent metabolic disturbance likely associated with intoxication or liver injury. Interestingly, inter-group correlations were notably lower, particularly between Group A and Groups B, F, and C, highlighting distinct metabolic profiles. Correlations between Group A samples and Group B samples were generally in the range of *r* = 0.85–0.87, indicating a metabolic shift due to the intervention. Similar moderate correlations were observed between the intervention groups (F, G) and the positive group (C), suggesting that fermented sour soup may have partially reversed or modulated the metabolic disruptions caused by the liver injury model.

#### 3.3.4 Metabolic pathways affected by fermented sour soup intervention

**FIGURE 12:**
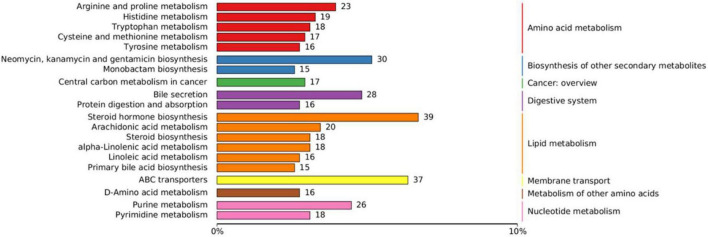
Metabolic pathway enrichment analysis bar plot (*p* < 0.05).

To interpret the biological relevance of the altered fecal metabolites associated with fermented sour soup intervention, pathway enrichment analysis was conducted. The bar plot (Figure 12) presents the statistically significant pathways, while the number of metabolites mapped to each pathway is shown for reference. The resulting bar plot illustrates the distribution and impact of significantly enriched metabolic pathways, categorized by biological function. Among the most prominently affected pathways were those involved in lipid metabolism, particularly steroid hormone biosynthesis (39 metabolites) and arachidonic acid metabolism (20 metabolites), highlighting a potential mechanism by which fermented sour soup exerts its liver-protective effects. These pathways are known to play critical roles in regulating inflammation, oxidative stress, and membrane stability, which are often disrupted during liver injury or alcohol-induced damage. Pathways related to membrane transport, especially the ABC transporters (37 metabolites), also showed substantial enrichment. This suggests that fermented sour soup may influence the transport and excretion of bile acids, xenobiotics, or endogenous metabolites mechanisms important for detoxification and metabolic homeostasis. Several amino acid metabolism pathways were enriched, including arginine and proline metabolism (23 metabolites), histidine metabolism (19), tryptophan metabolism (18), and cysteine and methionine metabolism (17). These findings point to altered amino acid utilization and nitrogen balance; processes often linked to gut-liver axis function and detoxification capacity. Additionally, digestive system-related pathways, such as bile secretion (28 metabolites) and protein digestion and absorption (16), were affected, suggesting that fermented sour soup modulates gastrointestinal activity and nutrient absorption and contribution to metabolic recovery. Enrichments in nucleotide metabolism (such as pyrimidine metabolism, 18 metabolites; purine metabolism, 26 metabolites) and biosynthesis of secondary metabolites (such as neomycin, kanamycin and gentamicin biosynthesis, 30 metabolites) indicate broader metabolic regulation possibly associated with microbial modulation in the gut following intervention.

#### 3.3.5 HMDB annotation of metabolites in response to fermented sour soup

**FIGURE 13:**
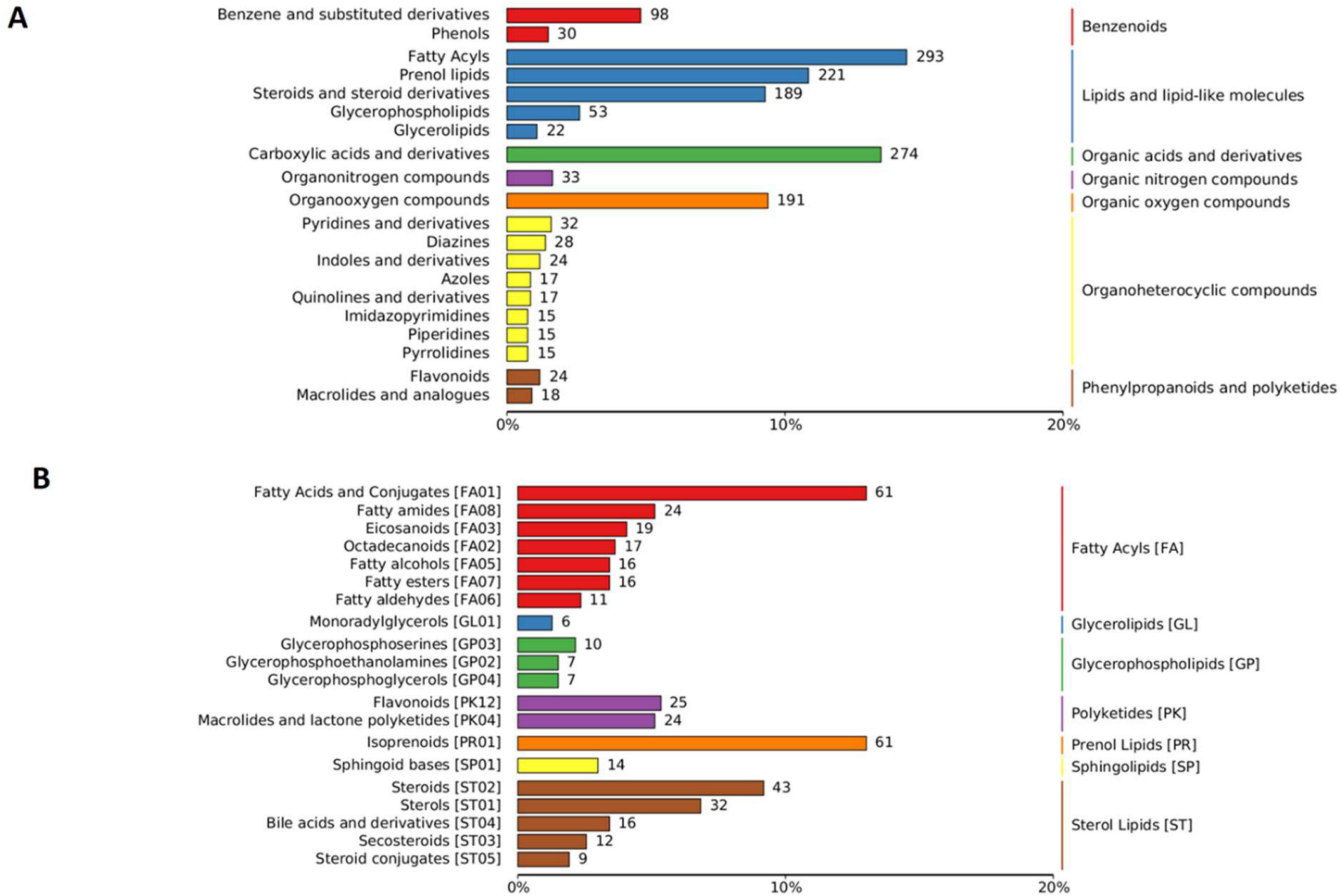
Metabolite annotation and classification using HMDB and LIPID MAPS. **(A).** HMDB annotation of metabolites. **(B)** LIPID MAPS database classification of metabolites (*p* < 0.05).

To further interpret the metabolic perturbations induced by fermented sour soup intervention, differential metabolites identified from fecal samples were annotated using the HMDB classification system. As shown in Figure 13A, the annotated metabolites were grouped into structural super classes and sub classes to elucidate chemical diversity and potential functional implications. Among the annotated classes, lipids and lipid-like molecules were overwhelmingly dominant, representing the most abundant superclass. Within this category, fatty acyls (*n* = 293) were the most prevalent, followed by prenol lipids (*n* = 221), steroids and steroid derivatives (*n* = 189), glycerophospholipids (*n* = 53), and glycerolipids (*n* = 22). These compounds are central to membrane composition, signaling pathways and energy metabolism, supporting detoxification capacity of liver and cellular repair processes which are key mechanisms underlying liver protection and recovery from alcohol-induced stress.

A substantial number of organic acids and derivatives (*n* = 274) were also identified. These metabolites are essential intermediates in central carbon metabolism, including the TCA cycle, amino acid catabolism and short-chain fatty acid production, all of which influence hepatic energy homeostasis and gut microbial activity. Organooxygen compounds (*n* = 191) and organonitrogen compounds (*n* = 33) were also enriched. The former may include alcohols, ketones and aldehydes involved in oxidative stress response, while the latter reflect nitrogen metabolism and microbial amino acid fermentation. A considerable number of benzenoids, including benzene and substituted derivatives (*n* = 98) and phenols (*n* = 30), were detected. These may derive from both diet and microbial aromatic compound metabolism and are known for their antioxidant, anti-inflammatory, and neuromodulatory roles. A diverse group of organonitrogen heterocycles, such as pyridines (*n* = 32), diazines (*n* = 28), indoles (*n* = 24), and quinolines (*n* = 17), along with flavonoids (*n* = 24) and macrolides (*n* = 18), represent a suite of bioactive secondary metabolites. Many of these are associated with microbial metabolism and may contribute to modulation of neurotransmission, oxidative balance, and immune responses.

#### 3.3.6 LIPID MAPS database classification of metabolites in mouse fecal samples following fermented sour soup intervention

The lipid metabolite profiling of mouse fecal samples, analyzed through the LIPID MAPS database, revealed a detailed subclass distribution indicative of the biochemical shifts associated with the hepatoprotective effects of fermented sour soup (Figure 13B). At the subclass level, the dominant categories included Fatty Acids and Conjugates (FA01) and Isoprenoids (PR01), each comprising 61 identified metabolites. These findings highlight the central role of fatty acid metabolism and isoprenoid biosynthesis in mediating the biological effects observed. Fatty acids, including saturated, unsaturated, and conjugated forms, are key energy substrates and signaling molecules that can influence liver detoxification pathways and alcohol metabolism. Isoprenoids, known for their involvement in cell membrane integrity and antioxidant functions, further underscore the metabolic modulation exerted by fermented sour soup.

Other prominent fatty acyl subclasses included Fatty amides (FA08) with 24 metabolites, Eicosanoids (FA03) at 19, Octadecanoids (FA02) at 17, Fatty alcohols (FA05) and Fatty esters (FA07) each with 16, and Fatty aldehydes (FA06) with 11. These subclasses play critical roles in inflammation regulation, cell signaling, and lipid peroxidation, suggesting an impact on oxidative stress and inflammatory processes. Within the lipid super classes, Glycerophospholipids showed notable diversity with 10 metabolites classified as Glycerophosphoserines (GP03), and 7 each for Glycerophosphoethanolamines (GP02) and Glycerophosphoglycerols (GP04). These phospholipids are essential components of cell membranes and are implicated in liver cell regeneration and membrane fluidity. Glycerolipids (GL) were represented by 6 Monoradylglycerols (GL01), reflecting potential changes in lipid storage and mobilization.

Among polyketides, the two main sub classes included Flavonoids (PK12) with 25 metabolites and Macrolides and lactone polyketides (PK04) with 24 metabolites. These bioactive compounds are well-known for their antioxidant, anti-inflammatory, and hepatoprotective properties, potentially contributing to the liver-protecting effects of fermented sour soup. The sterol lipid subclass encompassed multiple components, including Steroids (ST02) with 43 metabolites, Sterols (ST01) with 32, Bile acids and derivatives (ST04) at 16, Secosteroids (ST03) at 12, and Steroid conjugates (ST05) with 9 metabolites. These lipids are critically involved in cholesterol metabolism, bile acid synthesis, and liver function, aligning with the observed therapeutic effects. Finally, the Sphingolipids (SP01) sub class was represented by 14 Sphingoid bases, which are known to regulate cell apoptosis, proliferation, and inflammation, further suggesting modulation of liver health pathways.

#### 3.3.7 Differential metabolites analysis

**FIGURE 14:**
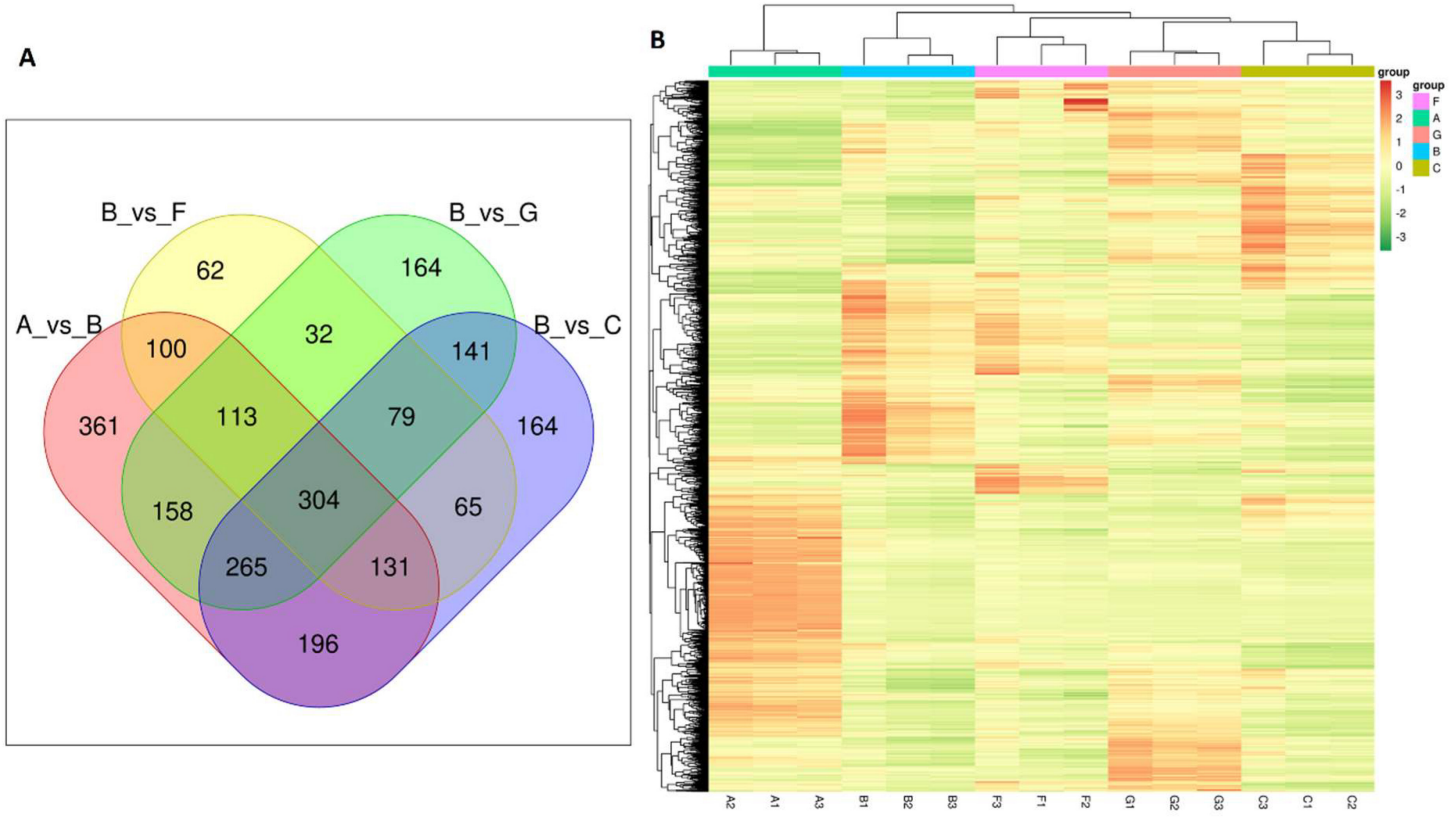
Fecal metabolome comparison and group-specific patterns via Venn diagram and heat maps. **(A)** Venn diagram showing the fecal metabolomic profiles across multiple comparison groups; **(B)** Heat maps of metabolites differentiation pattern among the groups (*n* = 3).

To investigate the metabolic impact of fermented sour soup, fecal metabolomic profiles were analyzed across multiple comparison groups (Figure 14A). A total of 3,151 metabolites were detected in each group. In the A vs. B comparison, representing the baseline difference, 1,628 differential metabolites were identified, including 646 upregulated and 982 downregulated metabolites. This comparison showed the highest number of differential metabolites, indicating a substantial shift in the metabolic profile. For intervention comparisons relative to the model group (Group B), 886 differential metabolites were identified in the B vs. F comparison, with 485 upregulated and 401 downregulated. The B vs. G comparison revealed 1,256 differential metabolites, of which 850 were upregulated and 406 downregulated, indicating a stronger metabolic response compared to Group F. In the B vs. C comparison, 1,345 differential metabolites were detected, with 618 upregulated and 727 downregulated, reflecting a balanced pattern of metabolic alterations. The heat map representing the metabolites differentiation pattern among the groups is presented in Figure 14B.

**FIGURE 15:**
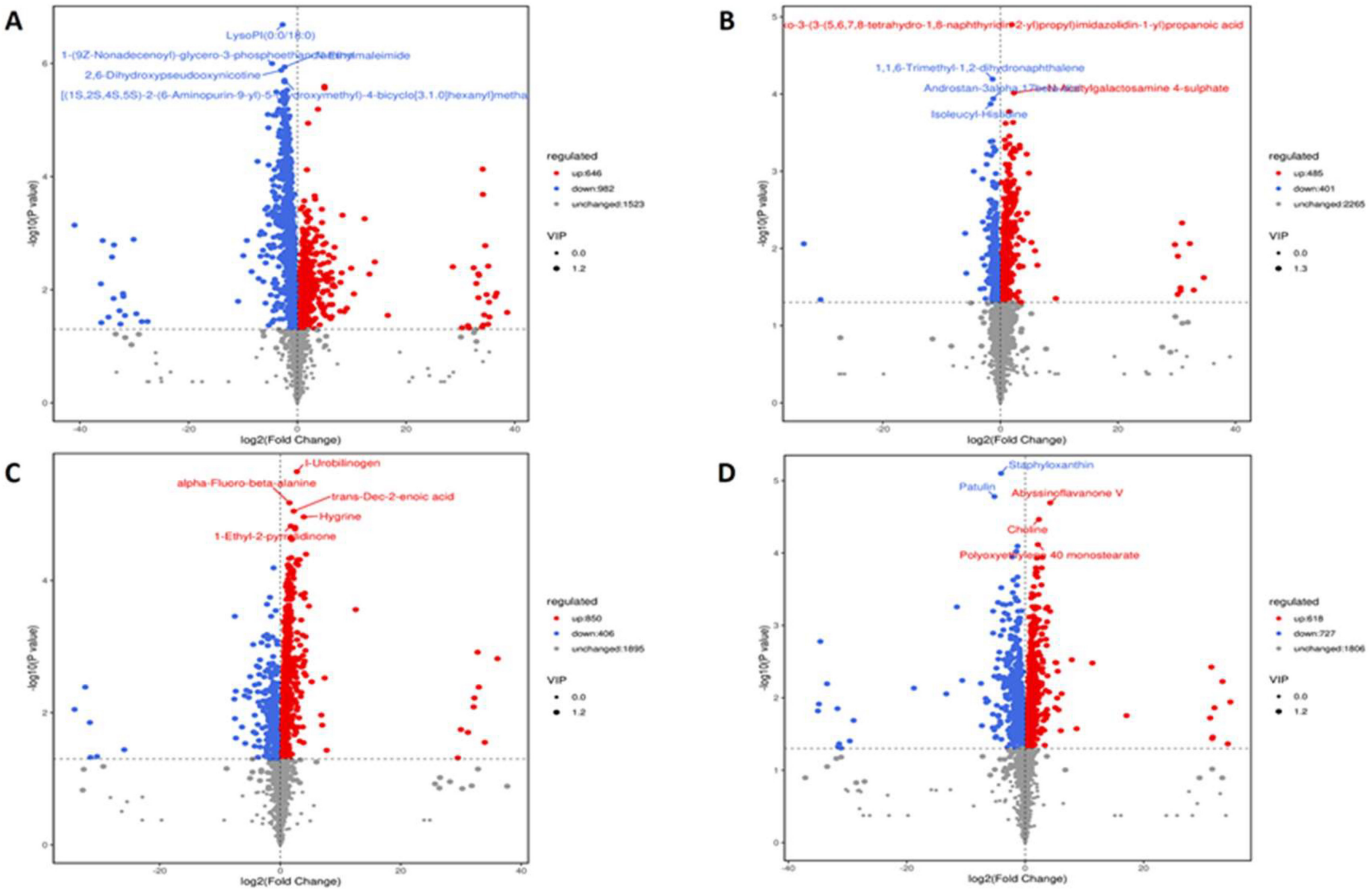
Volcano plot analysis between experimental groups. **(A)** Volcano plot analysis between group A (blank group) and group B (model group). **(B)** Volcano plot analysis between group B (model group) and group F (low dose group). **(C)** Volcano plot analysis between group B (model group) and group G (high dose group). **(D)** Volcano plot analysis between group B (model group) and group C (positive control group) (*n* = 3).

Volcano plot analysis was performed to identify significantly altered metabolites between group A (blank group) and group B (model group), revealing extensive metabolic changes induced by the model condition (Figure 15A). Among the total of 3,151 detected metabolites, 646 were significantly upregulated (shown in red), and 982 were significantly downregulated (shown in blue) in the model group, based on thresholds of | log*2* (fold change) | > 1 and *p*-value < 0.05. The remaining 1,523 metabolites (shown in gray) were not significantly altered. Several downregulated metabolites displayed both high fold changes and strong statistical significance, including LysoPI (0:0/18:0), 1-(9Z-Nonadecenoyl)-glycero-3-phosphoethanolamine, 2,6-Dihydroxypseudooxynicotine, and [(1S,2S,4S,5S)-2-(6-Aminopurin-9-yl)-5-(hydroxymethyl)-4-bicyclo [3.1.0] hexanyl] methanol. The size of the points reflects the variable importance in projection (VIP) values, indicating the relative contribution of each metabolite to the group discrimination.

The volcano plot reveals a clear metabolic shift in the model group compared to the low dose group, indicating that the condition modeled in group F significantly perturbed the metabolic profile (Figure 15B). Out of the total 3,151 detected metabolites, 485 were significantly upregulated (red), 401 were significantly downregulated (blue), and 2,265 remained unchanged (gray). Several metabolites exhibited both high fold changes and strong statistical significance, including upregulated compounds such as “2-oxo-3-(3-(5,6,7,8-tetrahydro-1,8-naphthyridin-2-yl)propyl)imidazolidin-1-yl)propanoic acid” and “Beta-N-Acetylgalactosamine 4-sulfate,” while key downregulated metabolites included “1,1,6-Trimethyl-1,2-dihydronaphthalene,” “Androstan-3alpha,17beta-diol,” and “Isoleucyl-Histidine.” Point size indicates VIP scores, reflecting the importance of each metabolite in distinguishing between groups. Volcano plot analysis between group B (model group) and group G (high dose group), revealing substantial metabolic changes induced by the high dose intervention (Figure 15C). Out of a total of 3,151 metabolites analyzed, 850 were significantly upregulated (red), 406 were significantly downregulated (blue), and 1,895 remained unchanged (gray). Noteworthy upregulated metabolites with high statistical significance included l-Urobilinogen, alpha-Fluoro-beta-alanine, trans-Dec-2-enoic acid, Hygrine, and 1-Ethyl-2-pyrrolidinone. These metabolites are located in the upper right quadrant of the plot, indicating strong upregulation in the high dose group.

Among the 3,151 analyzed metabolites, 618 were significantly upregulated (red), 727 were significantly downregulated (blue), and 1,806 showed no significant change (gray), based on thresholds of | log*2* (fold change) | > 1 and *p*-value < 0.05 in group B (model) vs. group C (positive control group) (Figure 15D). Key upregulated metabolites included Abyssinoflavanone V, Choline, and Polyoxyethylene1 40 monostearate, while notable downregulated compounds included Staphyloxanthin and Patulin, each exhibiting both high fold changes and strong statistical significance. The relative importance of each metabolite in discriminating between groups is illustrated by the size of the data points, which reflect Variable Importance in Projection (VIP) scores.

#### 3.3.8 Mechanistic insights into the hepatoprotective effects of fermented sour soup

**FIGURE 16:**
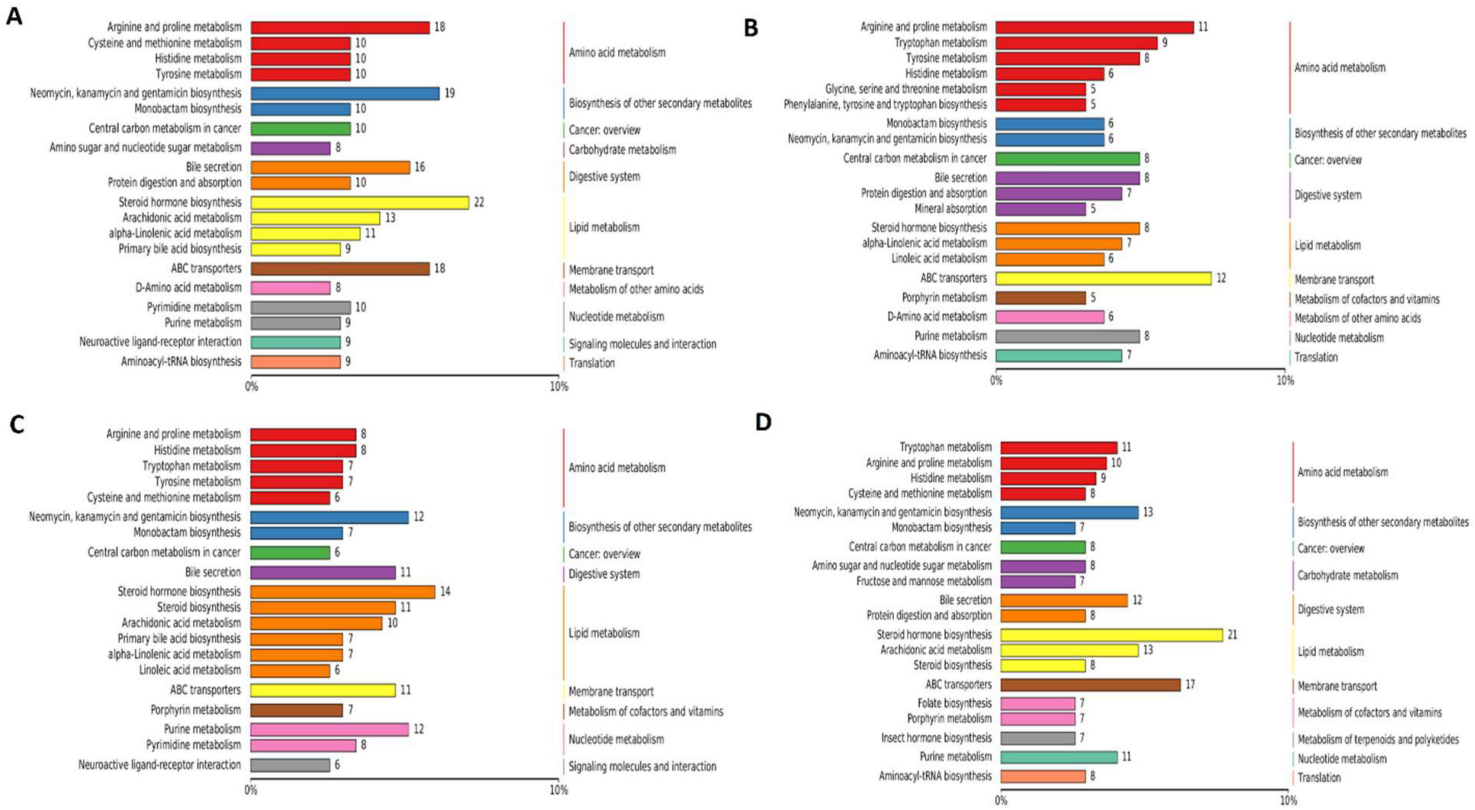
KEGG pathway annotation and enrichment analysis. **(A)** Comparison between groups A and B; **(B)** comparison between groups B and F. **(C)** comparison between groups B and G; **(D)** comparison between groups B and C (*p*-value < 0.05).

The KEGG pathway annotation and enrichment analyses revealed extensive metabolic remodeling induced by fermented sour soup across various experimental comparisons, particularly highlighting its influence on amino acid and lipid metabolism, microbial metabolite biosynthesis, and detoxification-related pathways. In the comparison between groups A and B, representing a blank versus model condition, fermented sour soup significantly modulated pathways involved in amino acid metabolism including arginine and proline, cysteine and methionine, histidine, and tyrosine metabolism as well as secondary metabolite biosynthesis, bile secretion, and protein digestion (Figure 16A). Lipid-related pathways such as steroid hormone biosynthesis, arachidonic acid metabolism, and alpha-linolenic acid metabolism were also enriched. The robust changes in arginine and proline metabolism (p ≈ 0.05) and high rich factors in steroid hormone biosynthesis and ABC transporters (∼1.3–1.5) highlight the strong regulatory impact of the intervention.

In the comparison between groups B and F, fermented sour soup continued to influence amino acid metabolism, particularly arginine and proline, tryptophan, tyrosine, and histidine pathways (Figure 16B). Moderate enrichment in microbial-derived secondary metabolite biosynthesis pathways and cancer metabolism, along with signaling pathways such as gap junction and circadian entrainment, was also observed. Dotplot analysis indicated high metabolite involvement in biosynthesis of phenylalanine, tyrosine, and tryptophan, suggesting enhanced detoxification, improved nutrient utilization, and modulation of neural signaling consistent with the proposed hepatoprotective effects of the intervention.

Further analysis comparing groups B and G revealed broad metabolic changes, especially in amino acid and lipid metabolism (Figure 16C). Notably enriched pathways included steroid hormone biosynthesis, sphingolipid metabolism, lipoic acid metabolism, and oxidative phosphorylation. Enrichment of neurotransmission-related pathways, such as dopaminergic synapse and endocrine-related signaling like aldosterone synthesis, along with associations with disease pathways (such as Parkinson’s and Chagas disease), indicate that fermented sour soup may impact membrane composition, hormonal regulation, and energy metabolism. Similarly, in the B vs. C comparison, significant enrichment was found in pathways related to amino acid metabolism, lipid biosynthesis, and cofactors such as folate and porphyrin metabolism (Figure 16D). Steroid hormone biosynthesis remained the most enriched, alongside ABC transporters, the AMPK signaling pathway, and several neuro-related disease pathways.

## 4 Discussion

The analysis of microbial composition across five intervention groups (A, B, C, F, G) revealed distinct patterns in both taxonomic structure and functional potential, suggesting that fermented sour soup elicits diverse microbiome responses depending on the intervention group. At the taxonomic level, microbial community composition was markedly influenced by intervention, with *Firmicutes* and *Bacteroidota* dominating across groups, albeit with higher relative abundance in group A. These two phyla are well-documented for their roles in energy harvest and fermentation of dietary fibers (39), suggesting that group A might possess an enhanced capacity for short-chain fatty acid (SCFA) production. At finer taxonomic levels, group-specific variations became more pronounced. For example, *Gammaproteobacteria* often associated with inflammation or dysbiosis were elevated in group C, while *Saccharimonadia* showed notable enrichment in groups F and G, suggesting potential shifts toward specialized fermentation or scavenging niches (40). At the genus level, the enrichment of *Muribaculum* in group B and greater genus-level diversity in group G suggest an intervention-modulated diversification of microbial functions, with potential implications for host-microbe interactions and gut resilience. The COG functional profiling corroborated the taxonomic findings, revealing functional specialization in response to intervention. At the phylum and genus levels, core metabolic processes such as amino acid and carbohydrate metabolism, energy production, and general function prediction dominated especially within Firmicutes, Bacteroidota, and genera such as Bacteroides, Lactobacillus, and Faecalibacterium. These findings align with known roles of these microbes in maintaining gut health and nutrient assimilation (41). Meanwhile, less characterized genera like Muribaculum showed involvement in general or unknown functions, hinting at potentially novel metabolic pathways might be influenced by the fermented sour soup.

A key finding emerged from the comparative COG functional analysis between intervention groups. Group A, which had the highest abundance of *Firmicutes* and *Limosilactobacillus*, also showed significant enrichment in genes involved in replication, transcription, signal transduction, and defense mechanisms. These functions are indicative of a dynamically growing and environmentally responsive microbiota (42). The elevated levels of cell motility and defense mechanisms further suggest that the gut microbiota in group A might be more robust in coping with environmental stressors introduced by the dietary intervention. In contrast, Group B demonstrated functional enrichment in amino acid transport, secondary metabolites biosynthesis, and energy production, indicating a potentially metabolically active microbiome specialized in processing dietary components and generating bioactive compounds. This functional profile was also associated with high abundance of Muribaculum, a genus increasingly linked to metabolic flexibility and host adaptation (43). Similarly, Group F showed a functional signature centered on translation, cell wall biogenesis, and carbohydrate metabolism, reflecting a microbiota oriented toward growth and structural maintenance. Further comparisons reinforced these patterns. Group C, with elevated *Gammaproteobacteria*, showed heightened activity in replication, defense mechanisms, and cell cycle control, potentially reflecting microbial stress responses or opportunistic adaptation. Its functional enrichment in RNA processing and chromatin structure suggests a microbiome which may contribute in genomic regulation and adaptation. In contrast, group F maintained a focus on basic metabolism and biosynthesis, indicating a less stress-reactive but growth-oriented community. KEGG pathway analysis at the class level substantiated these insights, showing dominant contributions from Clostridia, Bacteroidia, and Bacilli to pathways involved in carbohydrate and amino acid metabolism. The prominence of “Global and overview maps” across most classes indicates that core metabolic functions are conserved despite intervention variation, while differential representation of lipid metabolism, membrane transport, and xenobiotics degradation in taxa like Actinobacteria and Gammaproteobacteria suggests possible roles in intervention-specific metabolic adaptations. Additionally, classes such as Campylobacteria and Spirochaetia exhibited functions linked to environmental adaptation and motility, reinforcing the idea that fermented sour soup may contribute to modulate not only metabolic output but also ecological behavior of gut microbiota. Functionally, fermented sour soup appears to enhance microbial pathways that support host metabolism, gut barrier integrity, and stress resilience, while maintaining a low abundance of pathways associated with pathogenicity or drug resistance. The general increase in transcriptional, translational, and signal transduction functions across multiple groups suggests a microbiota is correlated with actively responding and adapting to dietary inputs.

The functional annotation of gut microbiota following fermented sour soup intervention revealed a complex and dynamic microbial landscape marked by conserved metabolic functions and intervention-specific adaptations. Across all taxonomic levels from phylum to species the “Global and overview maps” category consistently dominated KEGG pathway distributions, underscoring a universal reliance on core metabolic and biosynthetic pathways essential for microbial viability and host-microbe interactions (44). This conserved enrichment across diverse microbial taxa, including *Lachnospiraceae*, Ruminococcaceae, Bacteroidaceae, and Prevotellaceae, reflects the potential roles of these pathways in maintaining gut homeostasis and supporting host metabolism, particularly under dietary modulation by fermented sour soup. Functional enrichment in carbohydrate, amino acid, and energy metabolism was prominent across taxonomic ranks especially in dominant gut families and genera such as Lactobacillaceae, Muribaculaceae, Bacteroides, Lactobacillus, and Ruminococcus. These pathways are closely linked to microbial fermentation of dietary substrates, energy extraction, and the generation of short-chain fatty acids (SCFAs), which have been associated with hepatoprotective and anti-inflammatory effects (45). In parallel, taxa such as Enterobacteriaceae, Desulfovibrionaceae, Coriobacteriaceae, and genera like Clostridium and Desulfovibrio exhibited greater representation in pathways associated with lipid metabolism, membrane transport, and xenobiotics biodegradation. These functions are vital for modulating bile acid dynamics, breaking down alcohol-related compounds, and maintaining gut-liver axis integrity aligning with the liver-protective effects of the dietary intervention (46).

Comparative analyses between intervention and control groups revealed marked functional shifts in microbial community dynamics. Mice receiving fermented sour soup (Group C, G, F) consistently demonstrated enrichment in metabolic pathways, including carbohydrate, energy, and lipid metabolism (Group F: carbohydrate metabolism, *p* = 1.14e^–04^; lipid metabolism, *p* = 2.64e^–04^). These findings support the notion that fermented sour soup is associated with a microbiota conducive to energy harvesting and metabolic resilience, while potentially aiding detoxification and liver protection via increased excretory and xenobiotics-processing capabilities. Interestingly, while group B showed higher abundance in pathways related to immune and infectious disease responses (such as immune system, *p* = 3.93e^–05^; infectious diseases parasitic, *p* = 3.56e^–05^), this may contribute to immune-priming effect induced by bioactive compounds in fermented sour soup rather than pathogenic activation. Direct comparisons between intervention groups further elucidated the nuanced effects of fermented sour soup. Group F, in particular, exhibited robust enrichment in replication, repair, and nucleotide metabolism pathways (such as nucleotide metabolism, *p* = 5.20e^–06^), suggesting a microbiota with heightened proliferative and biosynthetic potential. This profile coincided with decreased representation of pathways associated with aging, neurodegeneration, and infection functions that were elevated in group B and C. Such results underscore the potential of fermented sour soup is correlated with a microbial community that is both metabolically active and protective against stress-related host dysfunctions. Moreover, the significant reduction in cancer-related and infectious disease pathways in group F, coupled with the increased abundance of digestion- and liver-related functions, strengthens the hypothesis that fermented sour soup may contribute to a healthier gut microbial ecosystem with implications for systemic health, particularly liver function.

The functional characterization of microbial communities using BugBase revealed distinct phenotypic and taxonomic shifts across sample groups, indicating variation in microbial ecology likely shaped by host-associated and environmental factors. Anaerobic phenotypes dominated across all groups, particularly in samples A, C, F, and G, consistent with the known anaerobic nature of gut microbiota (42). Taxa such as S24-7, Ruminococcaceae, Lachnospiraceae, and Clostridiales were key contributors, aligning with prior findings on their role in short-chain fatty acid production and host health (41). In contrast, sample B exhibited a unique microbial profile characterized by elevated aerobic, stress-tolerant, and pathogenic phenotypes, including a high abundance of *Enterobacteriaceae*, which are known for their mobile genetic elements and biofilm-forming capacity (47, 48). These features may confer increased pathogenic potential and antibiotic resistance, as supported by the co-occurrence of “Contains Mobile Elements” and “Forms Biofilms” phenotypes in B. Furthermore, differential abundance of Gram-negative taxa, especially in groups B and C, supports a functional divergence toward pathogenic or opportunistic lifestyles (49). Sample F was enriched in aerobic taxa like Lactobacillaceae, potentially reflecting environmental exposure or niche-specific oxygen availability (50). Functional enrichment analyses between comparative groups further highlighted consistent patterns such as group A was associated with fermentation and pathogen-related functions, while group B, C, and F exhibited increased gut-associated and environmental metabolic pathways, including nitrogen and sulfur cycling and phototrophy. These findings underscore the complexity of microbial functional dynamics and the importance of integrating phenotypic predictions with ecological context to better understand microbiome-associated processes in host and environmental systems.

The metabolic mechanisms underlying the hepatoprotective effects of fermented sour soup resulted robust alterations in fecal metabolite profiles and enriched biological pathways. A prominent finding was the significant enrichment of lipid metabolism pathways, particularly steroid hormone biosynthesis and arachidonic acid metabolism. The involvement of 39 and 20 differential metabolites, respectively, in these pathways suggests that fermented sour soup may contribute to modulate both endocrine and inflammatory processes. Steroid hormones are not only crucial for maintaining metabolic balance but also exert anti-inflammatory effects and contribute to hepatic regeneration following damage (51). Arachidonic acid and its derivatives play dual roles in inflammation and resolution; their regulated metabolism is essential for preventing chronic liver injury (52). The findings support the notion that fermented sour soup is associated with hepatoprotective effects via lipid-mediated anti-inflammatory and membrane-stabilizing mechanisms. The enrichment of ABC transporters and bile secretion pathways further suggests enhanced bile acid metabolism and improved detoxification capacity, which are critical for alcohol-induced liver injury recovery. ABC transporters mediate the efflux of bile salts, xenobiotics, and endogenous metabolites, and their upregulation has been associated with protection against hepatotoxins and maintenance of bile acid homeostasis (53). Amino acid metabolism was another major category of enrichment. Pathways including arginine and proline metabolism, histidine, tryptophan, tyrosine, cysteine, and methionine metabolism were significantly altered. These amino acids are key regulators of nitrogen balance, oxidative stress responses, neurotransmitter synthesis, and immune modulation. Arginine and proline, for instance, are involved in nitric oxide production and collagen synthesis, essential for tissue repair and vascular regulation (54). Tryptophan metabolism through the kynurenine pathway or serotonin synthesis also influences gut-brain communication (55). In addition, the enrichment of drug metabolism via cytochrome P450 and metabolism of xenobiotics by cytochrome P450 underscores an important mechanism of enhanced hepatic detoxification, likely contributing to reduced alcohol toxicity (56).

Chemical classification via HMDB annotation corroborated these findings by identifying a large proportion of differential metabolites as lipids and lipid-like molecules, including fatty acyls, steroids, and prenol lipids, all crucial for membrane integrity and hormone regulation. The presence of organic acids and derivatives, which are integral to the TCA cycle, short-chain fatty acid production, and amino acid catabolism, suggests enhanced energy metabolism and gut microbial activity both of which are essential for liver recovery. Additionally, detection of benzenoids, indoles, flavonoids, and organonitrogen heterocycles points to bioactive secondary metabolites, many of which are derived from microbial fermentation and are known to impact inflammation, oxidative balance, and neurotransmission (57, 58).

Despite the outstanding findings, this study has several limitations that should be considered when interpreting the findings. The relatively small sample sizes used for the multi-omics analyses limit statistical power and generalizability, reflecting the exploratory nature of the work and constraints in sample availability and analytical resources. Although rigorous data processing and conservative statistical approaches were applied, the results should be regarded as hypothesis-generating and require validation in larger cohorts. The metabolomics analysis was conducted using only negative ionization mode, which restricts metabolome coverage and may underrepresent metabolite classes that ionize preferentially in positive mode, such as amino acids and acylcarnitines; therefore, the conclusions are limited to the subset of detectable metabolites under the applied conditions. Functional predictions derived from 16S rRNA-based tools (such as PICRUSt2 and BugBase) are inherently inferential and lack direct experimental validation, necessitating cautious interpretation and further confirmation approaches such as shotgun metagenomics or qPCR. The use of a relatively low Pearson correlation threshold (| r| > 0.1) in network analysis may have introduced weak or potentially spurious associations, resulting in dense interaction networks that should not be over interpreted. The metabolite correlation network was not analyzed using a more stringent correlation threshold combined with formal statistical significance testing which may increase the possibility of false-positive findings and should therefore be interpreted with appropriate caution. Moreover, the evidence for direct liver injury was derived primarily from previously published studies rather than from newly conducted validation experiments within the present study. Finally, the compositional complexity of the fermented soup used in this study, which contains live microbes as well as diverse metabolites and plant-derived compounds, was not fully characterized, limiting the ability to disentangle the specific contributors to the observed effects. Collectively, these limitations highlight the need for more comprehensive and well-powered studies to validate and extend the present findings.

## 5 Conclusion

This study presents an integrated, in-depth analysis of the metabolic and microbiome-level responses to fermented sour soup intervention, revealing its multifaceted potential in supporting liver function, metabolic recovery and systemic health. Metabolomic profiling demonstrated significant modulation of key biochemical pathways, particularly those associated with lipid metabolism, amino acid turnover and detoxification, all of which are critical for maintaining hepatic resilience and counteracting alcohol-induced stress. These metabolic shifts were complemented by alterations in gut microbiota composition across taxonomic levels, with an increased abundance of beneficial taxa such as Lactobacillus, Bacteroides, Faecalibacterium and Ruminococcus, which were functionally enriched in pathways related to fermentation, energy harvesting, and xenobiotic biodegradation. The enrichment of microbial functions linked to bile acid metabolism, membrane transport, secondary metabolite biosynthesis, and immune modulation particularly in high-dose intervention groups supports the notion that fermented sour soup may contribute to exert its effects through the gut-liver axis, enhancing microbial plasticity and metabolic coordination. Furthermore, neuroprotective and endocrine-related pathways suggest potential contributions to systemic homeostasis. These findings indicate that fermented sour soup possibly acts as a potent dietary modulator capable of orchestrating metabolic and microbial adaptations to promote liver health, detoxification, and gut microbial resilience. Despite promising results in the mouse model, further clinical research is necessary to validate its translational relevance, elucidate precise molecular mechanisms, and assess long-term safety and efficacy in humans.

## Data Availability

The raw data generated in this study can be found in the NCBI (https://www.ncbi.nlm.nih.gov/) under accession PRJNA1470468.
